# Supplementary material for: Characterization of Seventeen Complete Mitochondrial Genomes: Structural Features and Phylogenetic Implications of the Lepidopteran Insects
Source: Insects. 2022 Oct 31;13(11):998. doi: 10.3390/insects13110998 (PMC9694843; doi:10.3390/insects13110998)
Supplement: Supplementary file 1 [file insects-13-00998-s001.zip › insects-1952476-supplementary.pdf]

Table S1.1 Mitogenomic characteristics of *Zaranga tukuringra*

Gene	Strand	Anti-codon	Start	Stop	Length	Start codon	End codon	Intergenic nucleotide
trnM(cau)	J	CAT	295	361	67			0
trnI(gau)	J	GAT	362	427	66			0
trnQ(uug)	N	TTG	425	493	69			-3
ND2	J		558	1559	1002	ATT	TAA	64
trnW(uca)	J	TCA	1564	1632	69			4
trnC(gca)	N	GCA	1625	1689	65			-8
trnY(gua)	N	GTA	1690	1755	66			0
COX1	J		1772	3307	1536	CGA	TAA	16
trnL2(uaa)	J	TAA	3303	3370	68			-5
COX2	J		3371	4052	682	ATG	T	0
trnK(cuu)	J	CTT	4053	4123	71			0
trnD(guc)	J	GTC	4153	4217	65			29
ATP8	J		4218	4379	162	ATT	TAA	0
ATP6	J		4373	5050	678	ATG	TAA	-7
COX3	J		5050	5838	789	ATG	TAA	-1
trnG(ucc)	J	TCC	5845	5909	65			6
ND3	J		5910	6263	354	ATT	TAA	0
trnA(ugc)	J	TGC	6271	6334	64			7
trnR(ucg)	J	TCG	6335	6397	63			0
trnN(guu)	J	GTT	6398	6462	65			0

trnS(gcu)	J	GCT	6465	6529	65			2
trnE(uuc)	J	TTC	6577	6642	66			47
trnF(gaa)	N	GAA	6644	6710	67			1
ND5	N		6710	8452	1743	ATT	TAA	-1
trnH(gug)	N	GTG	8453	8519	67			0
ND4	N		8519	9864	1346	ATT	TA(A)	-1
ND4L	N		9858	10145	288	ATG	TAA	-7
trnT(ugu)	J	TGT	10151	10215	65			5
trnP(ugg)	N	TGG	10216	10280	65			0
ND6	J		10289	10819	531	ATA	TAA	8
CYTB	J		10823	11974	1152	ATG	TAA	3
trnS2(uga)	J	TGA	11973	12038	66			-2
ND1	N		12057	12992	936	ATG	TAG	18
trnL(uag)	N	TAG	12994	13063	70			1
l-rRNA	N		13039	14411	1373			-25
trnV(uac)	N	TAC	14412	14479	68			0
s-rRNA	N		14480	15287	808			0

Table S1.2 Mitogenomic characteristics of *Gazalina chrysolopha*

Gene	Strand	Anti-codon	Start	Stop	Length	Start codon	End codon	Intergenic nucleotide
trnM(cau)	J	CAT	318	386	69			0
trnI(gau)	J	GAT	387	451	65			0
trnQ(uug)	N	TTG	449	517	69			-3
ND2	J		574	1587	1014	ATT	TAG	56
trnW(uca)	J	TCA	1592	1660	69			4
trnC(gca)	N	GCA	1653	1718	66			-8
trnY(gua)	N	GTA	1719	1783	65			0
COX1	J		1825	3360	1536	CGA	TAA	41
trnL2(uaa)	J	TAA	3356	3422	67			-5
COX2	J		3424	4125	702	ATG	TAA	1
trnK(cuu)	J	CTT	4106	4176	71			-20
trnD(guc)	J	GTC	4203	4276	74			26
ATP8	J		4277	4441	165	ATC	TAA	0
ATP6	J		4435	5112	678	ATG	TAA	-7
COX3	J		5116	5904	789	ATG	TAA	3
trnG(ucc)	J	TCC	5908	5973	66			3
ND3	J		5974	6327	354	ATT	TAA	0
trnA(ugc)	J	TGC	6354	6420	67			26
trnR(ucg)	J	TCG	6421	6483	63			0
trnN(guu)	J	GTT	6495	6559	65			11

trnS(gcu)	J	GCT	6563	6628	66			3
trnE(uuc)	J	TTC	6629	6694	66			0
trnF(gaa)	N	GAA	6694	6759	66			-1
ND5	N		6743	8503	1761	ATT	TAA	-17
trnH(gug)	N	GTG	8504	8569	66			0
ND4	N		8569	9908	1340	ATG	TAA	-1
ND4L	N		9914	10204	291	ATG	TAG	5
trnT(ugu)	J	TGT	10210	10274	65			5
trnP(ugg)	N	TGG	10275	10339	65			0
ND6	J		10347	10880	534	ATA	TAA	7
CYTB	J		10890	12041	1152	ATA	TAA	9
trnS2(uga)	J	TGA	12045	12113	69			3
ND1	N		12135	13070	936	GTG	TAA	21
trnL(uag)	N	TAG	13071	13142	72			0
l-rRNA	N		13167	14520	1354			24
trnV(uac)	N	TAC	14525	14592	68			4
s-rRNA	N		14593	15408	816			0

Table S1.3 Mitogenomic characteristics of *Deroca hyalina*

Gene	Strand	Anti-codon	Start	Stop	Length	Start codon	End codon	Intergenic nucleotide
trnM(cau)	J	CAT	371	438	68			0
trnI(gau)	J	GAT	439	505	67			0
trnQ(uug)	N	TTG	503	571	69			-3
ND2	J		643	1647	1005	ATT	TAA	71
trnW(uca)	J	TCA	1656	1722	67			8
trnC(gca)	N	GCA	1715	1778	64			-8
trnY(gua)	N	GTA	1779	1846	68			0
COX1	J		1846	3384	1539	TTG	TAA	-1
trnL2(uaa)	J	TAA	3380	3445	66			-5
COX2	J		3446	4162	717	ATA	TAA	0
trnK(cuu)	J	CTT	4128	4198	71			-35
trnD(guc)	J	GTC	4204	4271	68			5
ATP8	J		4272	4436	165	ATC	TAA	0
ATP6	J		4430	5107	678	ATG	TAA	-7
COX3	J		5107	5898	792	ATG	TAA	-1
trnG(ucc)	J	TCC	5901	5966	66			2
ND3	J		5967	6320	354	ATC	TAA	0
trnA(ugc)	J	TGC	6328	6392	65			7
trnR(ucg)	J	TCG	6393	6455	63			0
trnN(guu)	J	GTT	6460	6524	65			4

trnS(gcu)	J	GCT	6535	6596	62			10
trnE(uuc)	J	TTC	6593	6658	66			-4
trnF(gaa)	N	GAA	6666	6730	65			7
ND5	N		6714	8471	1758	ATA	TAA	-17
trnH(gug)	N	GTG	8472	8537	66			0
ND4	N		8538	9877	1340	ATG	TA(A)	0
ND4L	N		9884	10174	291	ATG	TAA	6
trnT(ugu)	J	TGT	10177	10240	64			2
trnP(ugg)	N	TGG	10241	10305	65			0
ND6	J		10308	10835	528	ATA	TAA	2
CYTB	J		10845	11993	1149	ATG	TAA	9
trnS2(uga)	J	TGA	11992	12057	66			-2
ND1	N		12075	13010	936	ATG	TAG	17
trnL(uag)	N	TAG	13012	13082	71			1
l-rRNA	N		13062	14452	1391			-21
trnV(uac)	N	TAC	14457	14525	69			4
s-rRNA	N		14526	15308	783			0

Table S1.4 Mitogenomic characteristics of *Menophra sp.*

Gene	Strand	Anti-codon	Start	Stop	Length	Start codon	End codon	Intergenic nucleotide
trnM(cau)	J	CAT	201	268	68			0
trnI(gau)	J	GAT	269	335	67			0
trnQ(uug)	N	TTG	349	417	69			13
ND2	J		481	1482	1002	ATA	TAA	63
trnW(uca)	J	TCA	1493	1562	70			10
trnC(gca)	N	GCA	1555	1620	66			-8
trnY(gua)	N	GTA	1621	1686	66			0
COX1	J		1692	3227	1536	CGA	TAA	5
trnL2(uaa)	J	TAA	3223	3289	67			-5
COX2	J		3290	3991	702	ATG	TAA	0
trnK(cuu)	J	CTT	3972	4041	70			-20
trnD(guc)	J	GTC	4042	4108	67			0
ATP8	J		4109	4273	165	ATA	TAA	0
ATP6	J		4267	4944	678	ATG	TAA	-7
COX3	J		4951	5739	789	ATG	TAA	6
trnG(ucc)	J	TCC	5753	5819	67			13
ND3	J		5820	6173	354	ATT	TAA	0
trnA(ugc)	J	TGC	6194	6257	64			20
trnR(ucg)	J	TCG	6258	6322	65			0
trnN(guu)	J	GTT	6328	6394	67			5

trnS(gcu)	J	GCT	6395	6460	66			0
trnE(uuc)	J	TTC	6467	6532	66			6
trnF(gaa)	N	GTT	6531	6596	66			-2
ND5	N		6580	8334	1755	ATT	TAA	-17
trnH(gug)	N	GTG	8335	8400	66			0
ND4	N		8404	9739	1336	ATG	T	3
ND4L	N		9745	10035	291	ATG	TAA	5
trnT(ugu)	J	TGT	10041	10105	65			5
trnP(ugg)	N	TGG	10106	10171	66			0
ND6	J		10174	10707	534	ATA	TAA	2
CYTB	J		10719	11876	1158	ATA	TAA	11
trnS2(uga)	J	TGA	11876	11943	68			-1
ND1	N		11962	12900	939	TTG	TAA	18
trnL(uag)	N	TAG	12901	12971	71			0
l-rRNA	N		12951	14362	1412			-21
trnV(uac)	N	TAC	14363	14428	66			0
s-rRNA	N		14429	15218	790			0

Table S1.5 Mitogenomic characteristics of *Dolbina paraexacta*

Gene	Strand	Anti-codon	Start	Stop	Length	Start codon	End codon	Intergenic nucleotide
trnM(cau)	J	CAT	289	357	69			0
trnI(gau)	J	GAT	358	422	65			0
trnQ(uug)	N	TTG	420	488	69			-3
ND2	J		541	1554	1014	ATT	TAA	52
trnW(uca)	J	TCA	1558	1624	67			3
trnC(gca)	N	GCA	1617	1680	64			-8
trnY(gua)	N	GTA	1681	1747	67			0
COX1	J		1756	3288	1533	AAA	TAA	8
trnL(uaa)	J	TAA	3284	3349	66			-5
COX2	J		3350	4031	682	ATG	T	0
trnK(cuu)	J	CTT	4032	4102	71			0
trnD(guc)	J	GTC	4105	4171	67			2
ATP8	J		4172	4333	162	ATC	TAA	0
ATP6	J		4327	5004	678	ATG	TAA	-7
COX3	J		5004	5795	792	ATG	TAA	-1
trnG(ucc)	J	TCC	5798	5863	66			2
ND3	J		5864	6217	354	ATT	TAA	0
trnA(ugc)	J	TGC	6220	6284	65			2
trnR(ucg)	J	TCG	6285	6351	67			0
trnN(guu)	J	GTT	6356	6422	67			4

trnS(gcu)	J	GCT	6425	6490	66			2
trnE(uuc)	J	TTC	6500	6566	67			9
trnF(gaa)	N	GTT	6565	6629	65			-2
ND5	N		6604	8352	1749	ATT	TAA	-26
trnH(gug)	N	GTG	8365	8431	67			12
ND4	N		8431	9765	1335	ATG	TAA	-1
ND4L	N		9766	10056	291	ATG	TAA	0
trnT(ugu)	J	TGT	10060	10125	66			3
trnP(ugg)	N	TGG	10125	10190	66			-1
ND6	J		10192	10722	531	ATG	TAA	1
CYTB	J		10723	11877	1155	ATG	TAA	0
trnS(uga)	J	TGA	11887	11952	66			9
ND1	N		11973	12908	936	GTG	TAA	20
trnL(uag)	N	TAG	12909	12975	67			0
l-rRNA	N		12956	14346	1391			-20
trnV(uac)	N	TAC	14348	14414	67			1
s-rRNA	N		14415	15191	777			0

Table S1.6 Mitogenomic characteristics of *Lassaba albidaria*

Gene	Strand	Anti-codon	Start	Stop	Length	Start codon	End codon	Intergenic nucleotide
trnM(cau)	J	CAT	165	232	68			0
trnI(gau)	J	GAT	233	299	67			0
trnQ(uug)	N	TTG	311	379	69			11
ND2	J		445	1446	1002	ATA	TAA	65
trnW(uca)	J	TCA	1461	1530	70			14
trnC(gca)	N	GCA	1523	1590	68			-8
trnY(gua)	N	GTA	1600	1665	66			9
COX1	J		1663	3207	1545	ATT	TAA	-3
trnL(uaa)	J	TAA	3203	3269	67			-5
COX2	J		3270	3971	702	ATG	TAA	0
trnK(cuu)	J	CTT	3952	4022	71			-20
trnD(guc)	J	GTC	4023	4088	66			0
ATP8	J		4089	4253	165	ATC	TAA	0
ATP6	J		4247	4924	678	ATG	TAA	-7
COX3	J		4937	5725	789	ATG	TAA	12
trnG(ucc)	J	TCC	5730	5795	66			4
ND3	J		5793	6149	357	ATA	TAA	-3
trnA(ugc)	J	TGC	6168	6235	68			18
trnR(ucg)	J	TCG	6236	6299	64			0
trnN(guu)	J	GTT	6300	6365	66			0

trnS(gcu)	J	GCT	6384	6449	66			18
trnE(uuc)	J	TTC	6450	6515	66			0
trnF(gaa)	N	GAA	6525	6591	67			9
ND5	N		6575	8329	1755	ATT	TAA	-17
trnH(gug)	N	GTG	8330	8396	67			0
ND4	N		8377	9735	1359	ATG	TAA	-20
ND4L	N		9737	10027	291	ATG	TAA	1
trnT(ugu)	J	TGT	10042	10105	64			14
trnP(ugg)	N	TGG	10106	10171	66			0
ND6	J		10174	10707	534	ATA	TAA	2
CYTB	J		10745	11896	1152	ATG	TAA	37
trnS(uga)	J	TGA	11895	11960	66			-2
ND1	N		11983	12927	945	ATA	TAA	22
trnL(uag)	N	TAG	12922	12989	68			-6
l-rRNA	N		13035	14413	1379			45
trnV(uac)	N	TAC	14408	14474	67			-6
s-rRNA	N		14475	15285	811			0

Table S1.7 Mitogenomic characteristics of *Sphragifera sigillata*

Gene	Strand	Anti-codon	Start	Stop	Length	Start codon	End codon	Intergenic nucleotide
trnM(cau)	J	CAT	1	68	68			0
trnI(gau)	J	GAT	71	137	67			2
trnQ(uug)	N	TTG	135	203	69			-3
ND2	J		258	1271	1014	ATT	TAA	54
trnW(uca)	J	TCA	1270	1337	68			-2
trnC(gca)	N	GCA	1330	1393	64			-8
trnY(gua)	N	GTA	1403	1466	64			9
COX1	J		1466	3004	1539	TTG	TAA	-1
trnL(uaa)	J	TAA	3000	3066	67			-5
COX2	J		3067	3748	682	ATG	T	0
trnK(cuu)	J	CTT	3749	3819	71			0
trnD(guc)	J	GTC	3828	3896	69			8
ATP8	J		3897	4058	162	ATA	TAA	0
ATP6	J		4052	4729	678	ATG	TAA	-7
COX3	J		4729	5517	789	ATG	TAA	-1
trnG(ucc)	J	TCC	5524	5591	68			6
ND3	J		5589	5945	357	ATA	TAA	-3
trnA(ugc)	J	TGC	5989	6059	71			43
trnR(ucg)	J	TCG	6062	6125	64			2
trnN(guu)	J	GTT	6135	6199	65			9

trnS(gcu)	J	GCT	6204	6269	66			4
trnE(uuc)	J	TTC	6270	6335	66			0
trnF(gaa)	N	GTT	6338	6403	66			2
ND5	N		6405	8141	1737	ATA	TAA	1
trnH(gug)	N	GTG	8156	8223	68			14
ND4	N		8223	9562	1340	ATG	TA(A)	-1
ND4L	N		9574	9864	291	ATG	TAA	11
trnT(ugu)	J	TGT	9867	9931	65			2
trnP(ugg)	N	TGG	9932	9997	66			0
ND6	J		10005	10535	531	ATT	TAA	7
CYTB	J		10546	11694	1149	ATA	TAA	10
trnS(uga)	J	TGA	11718	11784	67			23
ND1	N		11806	12744	939	ATG	TAA	21
trnL(uag)	N	TAG	12745	12815	71			0
l-rRNA	N		12825	13797	973			9
trnV(uac)	N	TAC	14197	14264	68			399
s-rRNA	N		14264	15103	840			-1

Table S1.8 Mitogenomic characteristics of *Asota tortuosa*

Gene	Strand	Anti-codon	Start	Stop	Length	Start codon	End codon	Intergenic nucleotide
trnM(cau)	J	CAT	289	357	69			0
trnI(gau)	J	GAT	358	422	65			0
trnQ(uug)	N	TTG	420	488	69			-3
ND2	J		543	1553	1011	ATA	TAA	54
trnW(uca)	J	TCA	1552	1618	67			-2
trnC(gca)	N	GCA	1611	1682	72			-8
trnY(gua)	N	GTA	1684	1747	64			1
COX1	J		1754	3289	1536	CGA	TAA	6
trnL(uaa)	J	TAA	3285	3351	67			-5
COX2	J		3352	4033	682	ATG	T	0
trnK(cuu)	J	CTT	4034	4103	70			0
trnD(guc)	J	GTC	4104	4168	65			0
ATP8	J		4169	4333	165	ATT	TAA	0
ATP6	J		4323	5000	678	ATG	TAG	-11
COX3	J		5008	5796	789	ATG	TAA	7
trnG(ucc)	J	TCC	5802	5868	67			5
ND3	J		5869	6222	354	ATT	TAA	0
trnA(ugc)	J	TGC	6257	6321	65			34
trnR(ucg)	J	TCG	6326	6390	65			4
trnN(guu)	J	GTT	6392	6456	65			1

trnS(gcu)	J	GCT	6469	6534	66			12
trnE(uuc)	J	TTC	6540	6605	66			5
trnF(gaa)	N	GAA	6604	6671	68			-2
ND5	N		6678	8423	1746	ATA	TAA	6
trnH(gug)	N	GTG	8424	8491	68			0
ND4	N		8491	9830	1340	ATG	TA(A)	-1
ND4L	N		9837	10124	288	ATG	TAA	6
trnT(ugu)	J	TGT	10130	10193	64			5
trnP(ugg)	N	TGG	10194	10259	66			0
ND6	J		10270	10794	525	ATT	TAA	10
CYTB	J		10824	11981	1158	ATG	TAA	29
trnS(uga)	J	TGA	11980	12044	65			-2
ND1	N		12069	13004	936	ATG	TAA	24
trnL(uag)	N	TAG	13006	13073	68			1
l-rRNA	N		13106	14493	1388			32
trnV(uac)	N	TAC	14495	14562	68			1
s-rRNA	N		14562	15370	809			-1

Table S1.9 Mitogenomic characteristics of *Olivenebula oberthueri*

Gene	Strand	Anti-codon	Start	Stop	Length	Start codon	End codon	Intergenic nucleotide
trnM(cau)	J	CAT	234	302	69			0
trnI(gau)	J	GAT	303	366	64			0
trnQ(uug)	N	TTG	364	432	69			-3
ND2	J		482	1495	1014	ATT	TAA	49
trnW(uca)	J	TCA	1494	1562	69			-2
trnC(gca)	N	GCA	1555	1619	65			-8
trnY(gua)	N	GTA	1636	1699	64			16
COX1	J		1702	3237	1536	CGA	TAA	2
trnL(uaa)	J	TAA	3233	3299	67			-5
COX2	J		3300	3981	682	ATG	T	0
trnK(cuu)	J	CTT	3982	4052	71			0
trnD(guc)	J	GTC	4063	4135	73			10
ATP8	J		4136	4297	162	ATA	TAA	0
ATP6	J		4291	4968	678	ATG	TAA	-7
COX3	J		4968	5756	789	ATG	TAA	-1
trnG(ucc)	J	TCC	5759	5823	65			2
ND3	J		5821	6177	357	ATA	TAA	-3
trnA(ugc)	J	TGC	6189	6255	67			11
trnR(ucg)	J	TCG	6257	6320	64			1
trnN(guu)	J	GTT	6321	6385	65			0

trnS(gcu)	J	GCT	6393	6458	66			7
trnE(uuc)	J	TTC	6459	6526	68			0
trnF(gaa)	N	GTT	6525	6591	67			-2
ND5	N		6602	8347	1746	ATT	TAA	10
trnH(gug)	N	GTG	8348	8413	66			0
ND4	N		8413	9752	1340	ATG	TA(A)	-1
ND4L	N		9786	10076	291	ATG	TAG	33
trnT(ugu)	J	TGT	10079	10143	65			2
trnP(ugg)	N	TGG	10144	10208	65			0
ND6	J		10216	10749	534	ATC	TAA	7
CYTB	J		10850	12001	1152	ATG	TAA	100
trnS(uga)	J	TGA	12016	12081	66			14
ND1	N		12099	13037	939	ATG	TAA	17
trnL(uag)	N	TAG	13039	13106	68			1
l-rRNA	N		13157	14510	1354			50
trnV(uac)	N	TAC	14511	14575	65			0
s-rRNA	N		14576	15389	814			0

Table S1.10 Mitogenomic characteristics of *Psyra falcipennis*

Gene	Strand	Anti-codon	Start	Stop	Length	Start codon	End codon	Intergenic nucleotide
trnM(cau)	J	CAT	376	441	66			0
trnI(gau)	J	GAT	442	509	68			0
trnQ(uug)	N	TTG	507	575	69			-3
ND2	J		647	1648	1002	ATA	TAA	71
trnW(uca)	J	TCA	1661	1728	68			12
trnC(gca)	N	GCA	1729	1795	67			0
trnY(gua)	N	GTA	1800	1865	66			4
COX1	J		1878	3413	1536	CGA	TAA	12
trnL(uaa)	J	TAA	3409	3475	67			-5
COX2	J		3476	4177	702	ATG	TAA	0
trnK(cuu)	J	CTT	4158	4228	71			-20
trnD(guc)	J	GTC	4228	4295	68			-1
ATP8	J		4296	4460	165	ATT	TAA	0
ATP6	J		4454	5131	678	ATG	TAA	-7
COX3	J		5154	5942	789	ATG	TAA	22
trnG(ucc)	J	TCC	5957	6022	66			14
ND3	J		6023	6376	354	ATT	TAA	0
trnA(ugc)	J	TGC	6393	6458	66			16
trnR(ucg)	J	TCG	6459	6524	66			0
trnN(guu)	J	GTT	6525	6590	66			0

trnS(gcu)	J	GCT	6591	6656	66			0
trnE(uuc)	J	TTC	6664	6730	67			7
trnF(gaa)	N	GAA	6729	6794	66			-2
ND5	N		6794	8535	1742	ATT	TA(A)	-1
trnH(gug)	N	GTG	8536	8601	66			0
ND4	N		8605	9940	1336	ATG	T	3
ND4L	N		9940	10230	291	ATG	TAA	-1
trnT(ugu)	J	TGT	10233	10297	65			2
trnP(ugg)	N	TGG	10298	10362	65			0
ND6	J		10365	10898	534	ATA	TAA	2
CYTB	J		10950	12101	1152	ATG	TAA	51
trnS(uga)	J	TGA	12101	12166	66			-1
ND1	N		12185	13120	936	TTG	TAA	18
trnL(uag)	N	TAG	13121	13189	69			0
l-rRNA	N		13210	14593	1384			20
trnV(uac)	N	TAC	14594	14658	65			0
s-rRNA	N		14659	15454	796			0

Table S1.11 Mitogenomic characteristics of *Numenes albofascia*

Gene	Strand	Anti-codon	Start	Stop	Length	Start codon	End codon	Intergenic nucleotide
trnM(cau)	J	CAT	379	446	68			0
trnI(gau)	J		453	519	67			6
trnQ(uug)	N	TTG	524	592	69			4
ND2	J		649	1662	1014	ATT	TAA	56
trnW(uca)	J	TCA	1668	1739	72			5
trnC(gca)	N	GCA	1732	1797	66			-8
trnY(gua)	N	GTA	1812	1879	68			14
COX1	J		1884	3419	1536	CGA	TAA	4
trnL(uaa)	J	TAA	3415	3482	68			-5
COX2	J		3483	4164	682	ATT	T	0
trnK(cuu)	J	CTT	4165	4235	71			0
trnD(guc)	J	GTC	4300	4366	67			64
ATP8	J		4367	4528	162	ATT	TAA	0
ATP6	J		4522	5199	678	ATG	TAA	-7
COX3	J		5199	5987	789	ATG	TAA	-1
trnG(ucc)	J	TCC	5990	6055	66			2
ND3	J		6056	6382	327	ATC	AAT	0
trnA(ugc)	J	TGC	6458	6524	67			75
trnR(ucg)	J	TCG	6586	6651	66			61
trnN(guu)	J	GTT	6652	6719	68			0

trnS(gcu)	J	GCT	6725	6793	69			5
trnE(uuc)	J	TTC	6795	6862	68			1
trnF(gaa)	N	GTT	6875	6941	67			12
ND5	N		6951	8693	1743	ATT	TAA	9
trnH(gug)	N	GTG	8694	8758	65			0
ND4	N		8758	10097	1340	ATG	TA(A)	-1
ND4L	N		10135	10425	291	ATG	TAA	37
trnT(ugu)	J	TGT	10431	10497	67			5
trnP(ugg)	N	TGG	10498	10562	65			0
ND6	J		10572	11102	531	ATA	TAA	9
CYTB	J		11110	12258	1149	ATA	TAA	7
trnS(uga)	J	TGA	12258	12324	67			-1
ND1	N		12376	13314	939	ATA	TAA	51
trnL(uag)	N	TAG	13315	13382	68			0
l-rRNA	N		13467	14837	1371			84
trnV(uac)	N	TAC	14839	14906	68			1
s-rRNA	N		14907	15756	850			0

Table S1.12 Mitogenomic characteristics of *Rhagastis albomarginatus*

Gene	Strand	Anti-codon	Start	Stop	Length	Start codon	End codon	Intergenic nucleotide
trnM(cau)	J	CAT	414	481	68			
trnI(gau)	J	GAT	483	547	65			1
trnQ(uug)	N	TTG	545	613	69			-3
ND2	J		667	1680	1014	ATT	TAA	53
trnW(uca)	J	TCA	1683	1749	67			2
trnC(gca)	N	GCA	1742	1805	64			-8
trnY(gua)	N	GTA	1806	1869	64			0
COX1	J		1879	3414	1536	CGA	TAA	9
trnL(uaa)	J	TAA	3410	3476	67			-5
COX2	J		3477	4156	680	ATG	TA(A)	0
trnK(cuu)	J	CTT	4159	4228	70			2
trnD(guc)	J	GTC	4229	4294	66			0
ATP8	J		4295	4456	162	ATC	TAA	0
ATP6	J		4450	5124	675	ATG	TAA	-7
COX3	J		5128	5919	792	ATG	TAA	3
trnG(ucc)	J	TCC	5922	5987	66			2
ND3	J		5988	6341	354	ATT	TAG	0
trnA(ugc)	J	TGC	6340	6405	66			-2
trnR(ucg)	J	TCG	6406	6470	65			0
trnN(guu)	J	GTT	6471	6537	67			0

trnS(gcu)	J	GCT	6538	6604	67			0
trnE(uuc)	J	TTC	6620	6685	66			15
trnF(gaa)	N	GAA	6686	6749	64			0
ND5*	N		6733	7332	600	ATT	TAA	-17
trnH(gug)	N	GTG	7384	7449	66			51
ND4	N		7455	8786	1332	ATG	TAA	5
ND4L	N		8788	9078	291	ATG	TAA	1
trnT(ugu)	J	TGT	9088	9153	66			9
trnP(ugg)	N	TGG	9153	9217	65			-1
ND6	J		9226	9756	531	ATG	TAA	8
CYTB	J		9756	10904	1149	ATG	TAA	-1
trnS(uga)	J	TGA	10911	10974	64			6
ND1	N		11003	11938	936	ATG	TAG	28
trnL(uag)	N	TAG	11940	12006	67			1
l-rRNA	N		11986	13356	1371			-21
trnV(uac)	N	TAC	13358	13422	65			1
s-rRNA	N		13423	14231	809			0

* Note: the position and length of ND5 may need further validation.

Table S1.13 Mitogenomic characteristics of *Dolbina inexacta*

Gene	Strand	Anti-codon	Start	Stop	Length	Start codon	End codon	Intergenic nucleotide
trnM(cau)	J	CAT	221	288	68			
trnI(gau)	J	GAT	289	353	65			0
trnQ(uug)	N	TTG	351	419	69			-3
ND2	J		472	1485	1014	ATT	TAA	52
trnW(uca)	J	TCA	1489	1555	67			3
trnC(gca)	N	GCA	1548	1611	64			-8
trnY(gua)	N	GTA	1612	1678	67			0
COX1	J		1687	3219	1533	AAA	TAA	8
trnL(uaa)	J	TAA	3215	3280	66			-5
COX2	J		3281	3962	682	ATG	T	0
trnK(cuu)	J	CTT	3963	4033	71			0
trnD(guc)	J	GTC	4036	4101	66			2
ATP8	J		4102	4263	162	ATC	TAA	0
ATP6	J		4257	4934	678	ATG	TAA	-7
COX3	J		4934	5725	792	ATG	TAA	-1
trnG(ucc)	J	TCC	5728	5794	67			2
ND3	J		5795	6148	354	ATT	TAA	0
trnA(ugc)	J	TGC	6151	6215	65			2
trnR(ucg)	J	TCG	6216	6282	67			0
trnN(guu)	J	GTT	6287	6352	66			4

trnS(gcu)	J	GCT	6355	6420	66			2
trnE(uuc)	J	TTC	6431	6497	67			10
trnF(gaa)	N	GAA	6496	6560	65			-2
ND5	N		6535	8283	1749	ATT	TAA	-26
trnH(gug)	N	GTG	8296	8362	67			22
ND4	N		8362	9696	1335	ATG	TAA	-1
ND4L	N		9697	9987	291	ATG	TAA	0
trnT(ugu)	J	TGT	9991	10056	66			3
trnP(ugg)	N	TGG	10056	10121	66			-1
ND6	J		10123	10653	531	ATG	TAA	1
CYTB	J		10654	11808	1155	ATG	TAA	0
trnS(uga)	J	TGA	11818	11883	66			9
ND1	N		11904	12839	936	GTG	TAA	20
trnL(uag)	N	TAG	12840	12906	67			0
l-rRNA	N		12887	14287	1401			-20
trnV(uac)	N	TAC	14289	14355	67			1
s-rRNA	N		14356	15167	812			0

Table S1.14 Mitogenomic characteristics of *Paralebeda femorata*

Gene	Strand	Anti-codon	Start	Stop	Length	Start codon	End codon	Intergenic nucleotide
trnM(cau)	J	CAT	280	348	69			
trnI(gau)	J	GAT	351	415	65			2
trnQ(uug)	N	TTG	435	503	69			19
ND2	J		552	1565	1014	ATA	TAA	48
trnW(uca)	J	TCA	1564	1631	68			-2
trnC(gca)	N	GCA	1624	1689	66			-8
trnY(gua)	N	GTA	1693	1758	66			3
COX1	J		1776	3314	1539	AAG	TAA	17
trnL(uaa)	J	TAA	3310	3376	67			-5
COX2	J		3377	4058	682	ATA	T	0
trnK(cuu)	J	CTT	4059	4129	71			0
trnD(guc)	J	GTC	4131	4199	69			1
ATP8	J		4200	4373	174	ATC	TAA	0
ATP6	J		4367	5044	678	ATG	TAA	-7
COX3	J		5054	5842	789	ATG	TAA	9
trnG(ucc)	J	TCC	5845	5913	69			2
ND3	J		5917	6267	351	ATT	TAA	3
trnA(ugc)	J	TGC	6268	6334	67			0
trnR(ucg)	J	TCG	6348	6414	67			13
trnN(guu)	J	GTT	6418	6483	66			3

trnS(gcu)	J	GCT	6492	6558	67			8
trnE(uuc)	J	TTC	6559	6626	68			0
trnF(gaa)	N	GTT	6628	6694	67			1
ND5	N		6703	8445	1743	ATT	TAG	8
trnH(gug)	N	GTG	8446	8511	66			0
ND4	N		8511	9850	1340	ATG	TA(A)	-1
ND4L	N		9905	10198	294	ATG	TAA	54
trnT(ugu)	J	TGT	10203	10267	65			4
trnP(ugg)	N	TGG	10268	10332	65			0
ND6	J		10335	10862	528	ATA	TAA	2
CYTB	J		10876	12024	1149	ATG	TAA	13
trnS(uga)	J	TGA	12023	12089	67			-2
ND1	N		12093	13043	951	ATG	TAG	3
trnL(uag)	N	TAG	13045	13113	69			1
l-rRNA	N		13121	14492	1372			7
trnV(uac)	N	TAC	14493	14557	65			0
s-rRNA	N		14558	15354	797			0

Table S1.15 Mitogenomic characteristics of *Zeuzera pyrina*

Gene	Strand	Anti-codon	Start	Stop	Length	Start codon	End codon	Intergenic nucleotide
trnM(cau)	J	CAT	322	388	67			
trnI(gau)	J	GAT	390	455	66			1
trnQ(uug)	N	TTG	453	521	69			-3
ND2	J		609	1622	1014	ATT	TAA	87
trnW(uca)	J	TCA	1621	1688	68			-2
trnC(gca)	N	GCA	1681	1746	66			-8
trnY(gua)	N	GTA	1759	1822	64			12
COX1	J		1828	3363	1536	CGA	TAA	5
trnL(uaa)	J	TAA	3359	3425	67			-5
COX2	J		3426	4142	717	ATG	TAA	0
trnK(cuu)	J	CTT	4108	4178	71			-35
trnD(guc)	J	GTC	4189	4261	73			10
ATP8	J		4262	4423	162	ATT	TAA	0
ATP6	J		4417	5094	678	ATG	TAA	-7
COX3	J		5094	5882	789	ATG	TAA	-1
trnG(ucc)	J	TCC	5885	5951	67			2
ND3	J		5952	6305	354	ATT	TAA	0
trnA(ugc)	J	TGC	6324	6388	65			18
trnR(ucg)	J	TCG	6389	6452	64			0
trnN(guu)	J	GTT	6453	6518	66			0

trnS(gcu)	J	GCT	6520	6585	66			1
trnE(uuc)	J	TTC	6596	6662	67			10
trnF(gaa)	N	GTT	6667	6732	66			4
ND5	N		6713	8470	1758	ATT	TAG	-20
trnH(gug)	N	GTG	8471	8536	66			0
ND4	N		8545	9885	1341	GTG	TAA	8
ND4L	N		9894	10175	282	ATG	TAA	8
trnT(ugu)	J	TGT	10178	10241	64			2
trnP(ugg)	N	TGG	10242	10306	65			0
ND6	J		10309	10839	531	ATC	TAA	2
CYTB	J		10843	11991	1149	ATG	TAA	3
trnS(uga)	J	TGA	11997	12065	69			5
ND1	N		12084	13022	939	ATG	TAA	18
trnL(uag)	N	TAG	13023	13090	68			0
l-rRNA	N		13070	14463	1394			-21
trnV(uac)	N	TAC	14437	14503	67			-27
s-rRNA	N		14506	15324	819			2

Table S1.16 Mitogenomic characteristics of *Phyllosphingia dissimilis*

Gene	Strand	Anti-codon	Start	Stop	Length	Start codon	End codon	Intergenic nucleotide
trnM(cau)	J	CAT	319	385	67			0
trnI(gau)	J	GAT	386	450	65			0
trnQ(uug)	N	TTG	448	516	69			-3
ND2	J		570	1583	1014	ATT	TAA	53
trnW(uca)	J	TCA	1582	1650	69			-2
trnC(gca)	N	GCA	1643	1706	64			-8
trnY(gua)	N	GTA	1708	1773	66			1
COX1	J		1771	3315	1545	ATT	TAA	-3
trnL(uaa)	J	TAA	3311	3378	68			-5
COX2	J		3379	4060	682	ATG	T	0
trnK(cuu)	J	CTT	4061	4131	71			0
trnD(guc)	J	GTC	4144	4208	65			12
ATP8	J		4209	4370	162	ATT	TAA	0
ATP6	J		4364	5041	678	ATG	TAA	-7
COX3	J		5041	5832	792	ATG	TAA	-1
trnG(ucc)	J	TCC	5839	5905	67			6
ND3	J		5906	6259	354	ATC	TAA	0
trnA(ugc)	J	TGC	6263	6328	66			3
trnR(ucg)	J	TCG	6329	6394	66			0
trnN(guu)	J	GTT	6395	6460	66			0

trnS(gcu)	J	GCT	6461	6526	66			0
trnE(uuc)	J	TTC	6527	6592	66			0
trnF(gaa)	N	GAA	6596	6664	69			3
ND5	N		6642	8387	1746	ATT	TAG	-23
trnH(gug)	N	GTG	8400	8467	68			12
ND4	N		8487	9824	1338	ATG	TAA	19
ND4L	N		9836	10126	291	ATG	TAA	11
trnT(ugu)	J	TGT	10132	10195	64			5
trnP(ugg)	N	TGG	10195	10260	66			-1
ND6	J		10272	10802	531	ATG	TAA	11
CYTB	J		10802	11950	1149	ATG	TAA	-1
trnS(uga)	J	TGA	11956	12028	73			5
ND1	N		12036	12983	948	ATG	TAG	7
trnL(uag)	N	TAG	12984	13050	67			0
l-rRNA	N		13030	14401	1372			-21
trnV(uac)	N	TAC	14403	14468	66			1
s-rRNA	N		14469	15245	777			0

Table S1.17 Mitogenomic characteristics of *Hyalinetta circumflexa*

Gene	Strand	Anti-codon	Start	Stop	Length	Start codon	End codon	Intergenic nucleotide
trnM(cau)	J	CAT	378	446	69			0
trnI(gau)	J	GAT	449	512	64			2
trnQ(uug)	N	TTG	510	578	69			-3
ND2	J		646	1659	1014	ATT	TAA	67
trnW(uca)	J	TCA	1662	1732	71			2
trnC(gca)	N	GCA	1725	1788	64			-8
trnY(gua)	N	GTA	1790	1856	67			1
COX1	J		1871	3406	1536	CGA	TAA	14
trnL(uaa)	J	TAA	3402	3470	69			-5
COX2	J		3471	4152	682	ATG	T	0
trnK(cuu)	J	CTT	4153	4223	71			0
trnD(guc)	J	GTC	4236	4304	69			12
ATP8	J		4305	4475	171	ATT	TAA	0
ATP6	J		4469	5146	678	ATG	TAA	-7
COX3	J		5159	5950	792	ATG	TAA	12
trnG(ucc)	J	TCC	6031	6097	67			80
ND3	J		6098	6451	354	ATT	TAA	0
trnA(ugc)	J	TGC	6460	6526	67			8
trnR(ucg)	J	TCG	6531	6595	65			4
trnN(guu)	J	GTT	6596	6661	66			0

trnS(gcu)	J	GCT	6679	6744	66			17
trnE(uuc)	J	TTC	6844	6913	70			99
trnF(gaa)	N	GTT	6927	6996	70			13
ND5	N		6980	8722	1743	ATC	TAA	-17
trnH(gug)	N	GTG	8735	8802	68			12
ND4	N		8805	10142	1338	ATG	TAA	2
ND4L	N		10152	10442	291	ATG	TAA	9
trnT(ugu)	J	TGT	10447	10512	66			4
trnP(ugg)	N	TGG	10512	10576	65			-1
ND6	J		10587	11123	537	ATG	TAA	10
CYTB	J		11123	12277	1155	ATG	TAA	-1
trnS(uga)	J	TGA	12276	12344	69			-2
ND1	N		12368	13303	936	ATG	TAA	23
trnL(uag)	N	TAG	13304	13374	71			0
l-rRNA	N		13354	14726	1373			-21
trnV(uac)	N	TAC	14727	14792	66			0
s-rRNA	N		14792	15576	785			-1

Table S2.1 The nucleotide composition of PCGs in *Gazalina chrysolopha*

Regions	Strand	Size (bp)	T(U)	C	A	G	AT(%)	GC(%)	GT(%)	AT skew	GC skew
PCGs	+	6924	38.4	17.8	33.8	10.1	72.2	27.9	48.5	-0.064	-0.278
PCGs	-	4326	47.3	7.2	28.5	17.1	75.8	24.3	64.4	-0.248	0.409
1st codon position	+	2308	31.3	15.7	37.2	15.8	68.5	31.5	47.1	0.087	0.001
1st codon position	-	1442	40.3	6.7	33.4	19.6	73.7	26.3	59.9	-0.094	0.489
2nd codon position	+	2308	45.9	19.4	22.5	12.2	68.4	31.6	58.1	-0.342	-0.228
2nd codon position	-	1442	51.1	12.2	21.1	15.6	72.2	27.8	66.7	-0.416	0.122
3rd codon position	+	2308	37.9	18.3	41.6	2.3	79.5	20.6	40.2	0.046	-0.781
3rd codon position	-	1442	50.4	2.6	31	16	81.4	18.6	66.4	-0.239	0.724
atp6	+	678	38.8	18.1	34.5	8.6	73.3	26.7	47.4	-0.058	-0.359
atp8	+	165	43	15.2	39.4	2.4	82.4	17.6	45.4	-0.044	-0.724
cox1	+	1536	36.1	19.1	30.6	14.3	66.7	33.4	50.4	-0.082	-0.145
cox2	+	702	34.8	18.4	36.2	10.7	71	29.1	45.5	0.02	-0.265
cox3	+	789	35.1	19.9	33	12	68.1	31.9	47.1	-0.032	-0.246
cytb	+	1152	37.8	18.4	33	10.8	70.8	29.2	48.6	-0.069	-0.262
nad1	-	936	47	8.1	25	19.9	72	28	66.9	-0.306	0.42
nad2	+	1014	44	14	35.4	6.6	79.4	20.6	50.6	-0.108	-0.359
nad3	+	354	42.1	16.9	34.2	6.8	76.3	23.7	48.9	-0.104	-0.429
nad4	-	1340	47.8	7	29.4	15.8	77.2	22.8	63.6	-0.238	0.386
nad4L	-	291	52.9	4.1	25.1	17.9	78	22	70.8	-0.357	0.625
nad5	-	1761	46.1	7.3	30.2	16.4	76.3	23.7	62.5	-0.208	0.386

nad6	+	534	40.4	17.2	36.5	5.8	76.9	23	46.2	-0.051	-0.496
rrnL	-	1354	40.8	4.9	39.6	14.8	80.4	19.7	55.6	-0.015	0.504
rrnS	-	816	39.7	4.8	44.2	11.3	83.9	16.1	51	0.054	0.405
rRNAs	-	2170	40.4	4.8	41.3	13.5	81.7	18.3	53.9	0.012	0.471
tRNAs	+	942	38.7	9.6	42.7	9	81.4	18.6	47.7	0.048	-0.029
tRNAs	-	537	38.9	6.1	40.8	14.2	79.7	20.3	53.1	0.023	0.394
Full genome	+	15474	36.4	16.1	39.6	8	76	24.1	44.4	0.043	-0.335

PCGs: protein-coding genes; +: major strand; -: minus strand

Table S2.2 The nucleotide composition of PCGs in *Deroca hyalina*

Regions	Strand	Size (bp)	T(U)	C	A	G	AT(%)	GC(%)	GT(%)	AT skew	GC skew
PCGs	+	6927	43.8	11.6	34.8	9.8	78.6	21.4	53.6	-0.114	-0.087
PCGs	-	4323	47.6	6.9	34.4	11	82	17.9	58.6	-0.16	0.229
1st codon position	+	2309	35.2	11.7	37.6	15.5	72.8	27.2	50.7	0.034	0.137
1st codon position	-	1441	40.7	6.2	37.8	15.4	78.5	21.6	56.1	-0.037	0.428
2nd codon position	+	2309	46.6	18.3	23	12.1	69.6	30.4	58.7	-0.338	-0.204
2nd codon position	-	1441	51.5	12.5	22	14	73.5	26.5	65.5	-0.401	0.058
3rd codon position	+	2309	49.6	4.9	43.8	1.7	93.4	6.6	51.3	-0.063	-0.474
3rd codon position	-	1441	50.7	2.1	43.6	3.7	94.3	5.8	54.4	-0.075	0.277
atp6	+	678	45	11.8	35.3	8	80.3	19.8	53	-0.121	-0.194
atp8	+	165	46.7	4.8	45.5	3	92.2	7.8	49.7	-0.013	-0.231
cox1	+	1539	41.8	13.3	30.9	14	72.7	27.3	55.8	-0.15	0.026
cox2	+	717	39.9	13	36.8	10.3	76.7	23.3	50.2	-0.04	-0.114
cox3	+	792	41.5	12.8	33.1	12.6	74.6	25.4	54.1	-0.113	-0.005
cytb	+	1149	42.3	12.6	34.9	10.2	77.2	22.8	52.5	-0.096	-0.107
nad1	-	936	47.8	7.6	31.3	13.4	79.1	21	61.2	-0.208	0.276
nad2	+	1005	48.9	9.7	35.7	5.8	84.6	15.5	54.7	-0.155	-0.252
nad3	+	354	48.6	9	35.3	7.1	83.9	16.1	55.7	-0.158	-0.123
nad4	-	1340	47.6	7.1	34.3	11	81.9	18.1	58.6	-0.163	0.218
nad4L	-	291	50.5	3.4	36.4	9.6	86.9	13	60.1	-0.162	0.474
nad5	-	1758	47	7	35.9	10	82.9	17	57	-0.134	0.177

nad6	+	528	46.2	8.5	40	5.3	86.2	13.8	51.5	-0.073	-0.233
rrnL	-	1391	41.4	4.9	44.3	9.4	85.7	14.3	50.8	0.034	0.317
rrnS	-	783	41.6	5.1	44.2	9.1	85.8	14.2	50.7	0.03	0.279
rRNAs	-	2174	41.5	5	44.3	9.3	85.8	14.3	50.8	0.032	0.303
tRNAs	+	924	38.7	9.2	41.9	10.2	80.6	19.4	48.9	0.039	0.05
tRNAs	-	537	39.1	5.4	43.8	11.7	82.9	17.1	50.8	0.056	0.37
Full genome	+	15332	41.2	10.7	40.2	7.9	81.4	18.6	49.1	-0.012	-0.153

Table S2.3 The nucleotide composition of PCGs in *Phyllosphingia dissimilis*

Regions	Strand	Size (bp)	T(U)	C	A	G	AT(%)	GC(%)	GT(%)	AT skew	GC skew
PCGs	+	6906	44.4	11.2	35.2	9.3	79.6	20.5	53.7	-0.116	-0.096
PCGs	-	4323	49.5	6	32.9	11.6	82.4	17.6	61.1	-0.202	0.316
1st codon position	+	2302	36.1	11.5	37.2	15.3	73.3	26.8	51.4	0.015	0.143
1st codon position	-	1441	42.1	5.9	35.9	16.2	78	22.1	58.3	-0.079	0.465
2nd codon position	+	2302	46.5	18.2	23.3	11.9	69.8	30.1	58.4	-0.332	-0.207
2nd codon position	-	1441	51.8	11.9	21.9	14.4	73.7	26.3	66.2	-0.407	0.098
3rd codon position	+	2302	50.5	4	45	0.5	95.5	4.5	51	-0.058	-0.769
3rd codon position	-	1441	54.8	0.3	40.9	4.1	95.7	4.4	58.9	-0.145	0.873
atp6	+	678	45.9	10.6	35.8	7.7	81.7	18.3	53.6	-0.123	-0.161
atp8	+	162	48.8	4.9	43.8	2.5	92.6	7.4	51.3	-0.053	-0.333
cox1	+	1545	40.2	14.5	32.2	13.1	72.4	27.6	53.3	-0.11	-0.052
cox2	+	682	42.1	11.1	36.7	10.1	78.8	21.2	52.2	-0.069	-0.048
cox3	+	792	42.2	12.2	33.2	12.4	75.4	24.6	54.6	-0.119	0.005
cytb	+	1149	43.5	11.7	35.1	9.7	78.6	21.4	53.2	-0.107	-0.089
nad1	-	948	49.5	7	30.6	13	80.1	20	62.5	-0.236	0.302
nad2	+	1014	49.6	9	35.8	5.6	85.4	14.6	55.2	-0.162	-0.23
nad3	+	354	48.6	9.6	35.6	6.2	84.2	15.8	54.8	-0.154	-0.214
nad4	-	1338	48.7	6.1	33.6	11.6	82.3	17.7	60.3	-0.183	0.314
nad4L	-	291	53.3	3.8	33	10	86.3	13.8	63.3	-0.235	0.45
nad5	-	1746	49.6	5.8	33.5	11.1	83.1	16.9	60.7	-0.194	0.308

nad6	+	531	48.6	7.3	39.7	4.3	88.3	11.6	52.9	-0.1	-0.258
rrnL	-	1372	41.7	5.1	42.9	10.3	84.6	15.4	52	0.014	0.34
rrnS	-	777	42.5	4.5	43.1	9.9	85.6	14.4	52.4	0.008	0.375
rRNAs	-	2149	42	4.9	43	10.2	85	15.1	52.2	0.012	0.352
tRNAs	+	939	39.3	9.4	41.6	9.7	80.9	19.1	49	0.029	0.017
tRNAs	-	535	42.1	4.9	40.2	12.9	82.3	17.8	55	-0.023	0.453
Full genome	+	15260	40.7	10.9	41	7.4	81.7	18.3	48.1	0.005	-0.191

Table S2.4 The nucleotide composition of PCGs in *Menophra sp.*

Regions	Strand	Size (bp)	T(U)	C	A	G	AT(%)	GC(%)	GT(%)	AT skew	GC skew
PCGs	+	6918	43.5	11.7	35	9.8	78.5	21.5	53.3	-0.108	-0.09
PCGs	-	4320	49	6.4	31.9	12.6	80.9	19	61.6	-0.211	0.324
1st codon position	+	2306	35.3	11.4	37	16.3	72.3	27.7	51.6	0.023	0.177
1st codon position	-	1440	43.1	5.8	34.4	16.7	77.5	22.5	59.8	-0.111	0.488
2nd codon position	+	2306	46.4	18.2	23.2	12.2	69.6	30.4	58.6	-0.334	-0.197
2nd codon position	-	1440	51.2	12.4	21.7	14.6	72.9	27	65.8	-0.404	0.08
3rd codon position	+	2306	48.7	5.6	44.8	0.9	93.5	6.5	49.6	-0.042	-0.722
3rd codon position	-	1440	52.8	1.1	39.6	6.5	92.4	7.6	59.3	-0.143	0.709
atp6	+	678	45.4	12.2	33.6	8.7	79	20.9	54.1	-0.149	-0.169
atp8	+	165	44.8	5.5	47.9	1.8	92.7	7.3	46.6	0.033	-0.5
cox1	+	1536	41.1	13.9	31.3	13.7	72.4	27.6	54.8	-0.136	-0.007
cox2	+	702	40	11.7	37.3	11	77.3	22.7	51	-0.035	-0.031
cox3	+	789	41.1	13.1	32.7	13.2	73.8	26.3	54.3	-0.113	0.005
cytb	+	1158	42.5	13.2	34.2	10.1	76.7	23.3	52.6	-0.108	-0.133
nad1	-	939	49.5	7.7	28.3	14.5	77.8	22.2	64	-0.272	0.308
nad2	+	1002	48.8	8.3	37.1	5.8	85.9	14.1	54.6	-0.136	-0.177
nad3	+	354	44.6	11.3	37.6	6.5	82.2	17.8	51.1	-0.086	-0.27
nad4	-	1336	48.2	6.5	32.3	12.9	80.5	19.4	61.1	-0.197	0.331
nad4L	-	291	53.6	3.4	32	11	85.6	14.4	64.6	-0.253	0.524
nad5	-	1755	48.7	6.2	33.5	11.6	82.2	17.8	60.3	-0.184	0.304

nad6	+	534	46.8	8.6	39.5	5.1	86.3	13.7	51.9	-0.085	-0.26
rrnL	-	1412	42.8	5	42.2	10.1	85	15.1	52.9	-0.007	0.34
rrnS	-	790	42.2	4.9	43.3	9.6	85.5	14.5	51.8	0.013	0.322
rRNAs	-	2202	42.6	5	42.6	9.9	85.2	14.9	52.5	0.001	0.333
tRNAs	+	937	37.4	9.6	43.3	9.7	80.7	19.3	47.1	0.074	0.006
tRNAs	-	536	41	5	42.2	11.8	83.2	16.8	52.8	0.013	0.4
Full genome	+	15250	39.9	11.4	41	7.8	80.9	19.2	47.7	0.013	-0.189

Table S2.5 The nucleotide composition of PCGs in *Numenes albofascia*

Regions	Strand	Size (bp)	T(U)	C	A	G	AT(%)	GC(%)	GT(%)	AT skew	GC skew
PCGs	+	5187	40.8	15	33.9	10.3	74.7	25.3	51.1	-0.092	-0.187
PCGs	-	4311	48.1	6.6	30.4	15	78.5	21.6	63.1	-0.226	0.39
1st codon position	+	1729	32.9	14.3	37	15.7	69.9	30	48.6	0.059	0.046
1st codon position	-	1437	41.8	6.1	33.7	18.5	75.5	24.6	60.3	-0.107	0.507
2nd codon position	+	1729	45.5	19.2	22.5	12.8	68	32	58.3	-0.338	-0.199
2nd codon position	-	1437	51.8	12.1	20.7	15.4	72.5	27.5	67.2	-0.43	0.119
3rd codon position	+	1729	44	11.5	42.3	2.3	86.3	13.8	46.3	-0.02	-0.671
3rd codon position	-	1437	50.7	1.5	36.7	11	87.4	12.5	61.7	-0.16	0.756
atp6	+	678	40.7	15.9	35	8.4	75.7	24.3	49.1	-0.076	-0.309
atp8	+	162	46.3	11.1	40.7	1.9	87	13	48.2	-0.064	-0.714
cox1	+	1536	37.9	17	31.2	13.9	69.1	30.9	51.8	-0.097	-0.099
cox2	+	682	37.1	16	36.2	10.7	73.3	26.7	47.8	-0.012	-0.198
cox3	+	789	38.4	15.5	33.2	12.9	71.6	28.4	51.3	-0.073	-0.089
nad1	-	939	47.3	7.3	27.9	17.5	75.2	24.8	64.8	-0.258	0.408
nad2	+	1014	47.4	11.1	35.4	6	82.8	17.1	53.4	-0.145	-0.299
nad3	+	327	45	14.4	33.6	7	78.6	21.4	52	-0.144	-0.343
nad4	-	1340	47.8	6.8	31.1	14.3	78.9	21.1	62.1	-0.211	0.357
nad4L	-	291	52.9	3.4	29.2	14.4	82.1	17.8	67.3	-0.289	0.615
nad5	-	1743	48	6.5	31.3	14.2	79.3	20.7	62.2	-0.21	0.372
rrnL	-	1371	41.9	4.7	41.6	11.7	83.5	16.4	53.6	-0.004	0.425

rrnS	-	850	40.7	4.5	44.7	10.1	85.4	14.6	50.8	0.047	0.387
rRNAs	-	2221	41.5	4.6	42.8	11.1	84.3	15.7	52.6	0.015	0.411
tRNAs	+	682	38.4	9.1	42.5	10	80.9	19.1	48.4	0.051	0.046
tRNAs	-	536	39.4	4.9	43.5	12.3	82.9	17.2	51.7	0.05	0.435
Full genome	+	15756	38.6	13.5	40.3	7.6	78.9	21.1	46.2	0.021	-0.281
Regions	Strand	Size (bp)	T(U)	C	A	G	AT(%)	GC(%)	GT(%)	AT skew	GC skew
PCGs	+	5187	40.8	15	33.9	10.3	74.7	25.3	51.1	-0.092	-0.187

Table S2.6 The nucleotide composition of PCGs in *Rhagastis albomarginatus*

Regions	Strand	Size (bp)	T(U)	C	A	G	AT(%)	GC(%)	GT(%)	AT skew	GC skew
PCGs	+	6891	42.5	12.4	35.6	9.5	78.1	21.9	52	-0.088	-0.13
PCGs	-	3159	48.2	6	33	12.8	81.2	18.8	61	-0.188	0.366
1st codon position	+	2297	35.4	11.8	37.7	15.2	73.1	27	50.6	0.032	0.126
1st codon position	-	1053	42.2	6.4	35.4	16	77.6	22.4	58.2	-0.087	0.432
2nd codon position	+	2297	46.1	18.6	23.2	12.1	69.3	30.7	58.2	-0.332	-0.214
2nd codon position	-	1053	51	10.8	22.7	15.5	73.7	26.3	66.5	-0.384	0.177
3rd codon position	+	2297	45.9	6.7	46	1.4	91.9	8.1	47.3	0.001	-0.658
3rd codon position	-	1053	51.6	0.7	40.8	6.9	92.4	7.6	58.5	-0.116	0.825
atp6	+	675	43	13.6	35.7	7.7	78.7	21.3	50.7	-0.092	-0.278
atp8	+	162	45.7	9.3	43.2	1.9	88.9	11.2	47.6	-0.028	-0.667
cox1	+	1536	38.7	15.4	32.4	13.5	71.1	28.9	52.2	-0.088	-0.063
cox2	+	680	39.1	12.9	37.8	10.1	76.9	23	49.2	-0.017	-0.121
cox3	+	792	40.5	13	33.8	12.6	74.3	25.6	53.1	-0.09	-0.015
cytb	+	1149	42	13.2	34.7	10	76.7	23.2	52	-0.095	-0.139
nad1	-	936	47.6	7.5	30.3	14.5	77.9	22	62.1	-0.222	0.32
nad2	+	1014	48.6	8.7	36.8	5.9	85.4	14.6	54.5	-0.139	-0.189
nad3	+	354	44.9	10.5	37.6	7.1	82.5	17.6	52	-0.089	-0.194
nad4	-	1332	48	6.6	33	12.5	81	19.1	60.5	-0.186	0.307
nad4L	-	291	53.6	3.4	31.3	11.7	84.9	15.1	65.3	-0.263	0.545
nad5	-	600*	47.2	3.3	38	11.5	85.2	14.8	58.7	-0.108	0.551

nad6	+	531	46.7	7.9	40.7	4.7	87.4	12.6	51.4	-0.069	-0.254
rrnL	-	1371	41.6	5	42.4	10.9	84	15.9	52.5	0.009	0.37
rrnS	-	809	41.8	4.7	42.5	11	84.3	15.7	52.8	0.009	0.402
rRNAs	-	2180	41.7	4.9	42.4	11	84.1	15.9	52.7	0.009	0.382
tRNAs	+	957	39.5	9.3	41.8	9.4	81.3	18.7	48.9	0.028	0.006
tRNAs	-	524	40.6	5.2	41.2	13	81.8	18.2	53.6	0.007	0.432
Full genome	+	14231	40.5	11.7	40.3	7.5	80.8	19.2	48	-0.003	-0.217

*Note: The ND5 gene may need further validation.

Table S2.7 The nucleotide composition of PCGs in *Zeuzera pyrina*

Regions	Strand	Size (bp)	T(U)	C	A	G	AT(%)	GC(%)	GT(%)	AT skew	GC skew
PCGs	+	6930	42	14.3	33.6	10	75.6	24.3	52	-0.111	-0.177
PCGs	-	4320	47.9	6.7	30.9	14.4	78.8	21.1	62.3	-0.216	0.363
1st codon position	+	2310	33.6	13.6	37	15.8	70.6	29.4	49.4	0.048	0.076
1st codon position	-	1440	41.9	6.2	33.7	18.1	75.6	24.3	60	-0.109	0.487
2nd codon position	+	2310	46	19.3	22.3	12.5	68.3	31.8	58.5	-0.347	-0.214
2nd codon position	-	1440	51.7	12.4	20.2	15.6	71.9	28	67.3	-0.438	0.114
3rd codon position	+	2310	46.5	10.1	41.6	1.7	88.1	11.8	48.2	-0.056	-0.708
3rd codon position	-	1440	50.1	1.5	38.8	9.5	88.9	11	59.6	-0.127	0.723
atp6	+	678	43.2	15.2	32.4	9.1	75.6	24.3	52.3	-0.142	-0.248
atp8	+	162	50	6.2	42	1.9	92	8.1	51.9	-0.087	-0.538
cox1	+	1536	39.4	16.1	30.8	13.7	70.2	29.8	53.1	-0.122	-0.083
cox2	+	717	38.4	15.6	35.3	10.7	73.7	26.3	49.1	-0.042	-0.185
cox3	+	789	37.4	16.9	32.3	13.4	69.7	30.3	50.8	-0.073	-0.113
cytb	+	1149	41.3	14.5	34	10.1	75.3	24.6	51.4	-0.097	-0.18
nad1	-	939	46	7.8	29.3	16.9	75.3	24.7	62.9	-0.222	0.371
nad2	+	1014	47.9	10.8	34.9	6.3	82.8	17.1	54.2	-0.157	-0.264
nad3	+	354	43.8	15.3	34.2	6.8	78	22.1	50.6	-0.123	-0.385
nad4	-	1341	47.4	6.9	31.5	14.2	78.9	21.1	61.6	-0.202	0.343
nad4L	-	282	51.4	3.9	30.1	14.5	81.5	18.4	65.9	-0.261	0.577
nad5	-	1758	48.8	6.5	31.5	13.3	80.3	19.8	62.1	-0.216	0.343

nad6	+	531	46.7	10.5	36.7	6	83.4	16.5	52.7	-0.12	-0.273
rrnL	-	1394	41.5	4.7	42.1	11.6	83.6	16.3	53.1	0.007	0.421
rrnS	-	819	40.3	4.6	43.8	11.2	84.1	15.8	51.5	0.042	0.415
rRNAs	-	2213	41.1	4.7	42.7	11.5	83.8	16.2	52.6	0.02	0.419
tRNAs	+	940	40.3	9.5	41	9.3	81.3	18.8	49.6	0.008	-0.011
tRNAs	-	531	40.1	5.5	40.5	13.9	80.6	19.4	54	0.005	0.437
Full genome	+	15324	39.1	13.3	39.8	7.9	78.9	21.2	47	0.009	-0.256

Table S2.8 The nucleotide composition of PCGs in *Psyra falcipennis*

Regions	Strand	Size (bp)	T(U)	C	A	G	AT(%)	GC(%)	GT(%)	AT skew	GC skew
PCGs	+	6912	42.6	11.5	35.8	10.1	78.4	21.6	52.7	-0.087	-0.067
PCGs	-	4302	49.5	6.5	31.5	12.5	81	19	62	-0.222	0.315
1st codon position	+	2304	35.4	10.9	37.3	16.4	72.7	27.3	51.8	0.026	0.201
1st codon position	-	1434	41.8	5.9	35.4	16.9	77.2	22.8	58.7	-0.084	0.48
2nd codon position	+	2304	45.9	18.4	23.3	12.4	69.2	30.8	58.3	-0.327	-0.195
2nd codon position	-	1434	51.1	12.6	21.6	14.7	72.7	27.3	65.8	-0.406	0.079
3rd codon position	+	2304	46.5	5.3	46.8	1.5	93.3	6.8	48	0.003	-0.561
3rd codon position	-	1434	55.6	1	37.7	5.8	93.3	6.8	61.4	-0.192	0.711
atp6	+	678	42.3	12.7	36.3	8.7	78.6	21.4	51	-0.077	-0.186
atp8	+	165	45.5	6.1	45.5	3	91	9.1	48.5	0	-0.333
cox1	+	1536	39.3	13.7	32.9	14.1	72.2	27.8	53.4	-0.088	0.014
cox2	+	702	39.7	11.7	37.7	10.8	77.4	22.5	50.5	-0.026	-0.038
cox3	+	789	40.8	12.9	33.3	12.9	74.1	25.8	53.7	-0.101	0
cytb	+	1152	41.9	12.4	35.5	10.2	77.4	22.6	52.1	-0.083	-0.1
nad1	-	936	49.7	7.7	28.1	14.5	77.8	22.2	64.2	-0.277	0.308
nad2	+	1002	48.2	8.9	36.5	6.4	84.7	15.3	54.6	-0.138	-0.163
nad3	+	354	46.9	10.2	36.2	6.8	83.1	17	53.7	-0.129	-0.2
nad4	-	1336	49.6	6.4	32.1	11.8	81.7	18.2	61.4	-0.214	0.295
nad4L	-	291	54	3.4	30.6	12	84.6	15.4	66	-0.276	0.556
nad5	-	1742	48.6	6.4	33.1	11.9	81.7	18.3	60.5	-0.19	0.302

nad6	+	534	46.1	7.1	40.6	6.2	86.7	13.3	52.3	-0.063	-0.07
rrnL	-	1384	43.3	5.1	41.5	10	84.8	15.1	53.3	-0.02	0.324
rrnS	-	796	43.5	4.6	42.3	9.5	85.8	14.1	53	-0.013	0.345
rRNAs	-	2180	43.3	5	41.8	9.9	85.1	14.9	53.2	-0.018	0.331
tRNAs	+	936	37.7	9	43.9	9.4	81.6	18.4	47.1	0.076	0.023
tRNAs	-	533	40.5	5.1	42.8	11.6	83.3	16.7	52.1	0.027	0.393
Full genome	+	15489	39.3	11.1	41.8	7.8	81.1	18.9	47.1	0.031	-0.175

Table S2.9 The nucleotide composition of PCGs in *Zaranga tukuringra*

Regions	Strand	Size (bp)	T(U)	C	A	G	AT(%)	GC(%)	GT(%)	AT skew	GC skew
PCGs	+	6885	40.4	14.7	35	9.9	75.4	24.6	50.3	-0.072	-0.196
PCGs	-	4311	49.5	6.5	30.1	13.9	79.6	20.4	63.4	-0.244	0.366
1st codon position	+	2295	32.3	14.2	37.7	15.8	70	30	48.1	0.077	0.054
1st codon position	-	1437	42.2	5.9	34.8	17.1	77	23	59.3	-0.096	0.486
2nd codon position	+	2295	46.1	19.1	22.6	12.2	68.7	31.3	58.3	-0.341	-0.218
2nd codon position	-	1437	51.6	12.4	20.9	15.1	72.5	27.5	66.7	-0.424	0.099
3rd codon position	+	2295	42.8	10.9	44.6	1.7	87.4	12.6	44.5	0.021	-0.737
3rd codon position	-	1437	54.7	1.1	34.6	9.6	89.3	10.7	64.3	-0.225	0.792
atp6	+	678	39.8	17.1	35.1	8	74.9	25.1	47.8	-0.063	-0.365
atp8	+	162	45.7	8	43.2	3.1	88.9	11.1	48.8	-0.028	-0.444
cox1	+	1536	38.6	16.6	30.7	14.1	69.3	30.7	52.7	-0.114	-0.083
cox2	+	682	38.6	14.4	36.8	10.3	75.4	24.7	48.9	-0.023	-0.167
cox3	+	789	36.2	16.5	34.1	13.2	70.3	29.7	49.4	-0.031	-0.111
cytb	+	1152	38.3	16.2	34.9	10.6	73.2	26.8	48.9	-0.046	-0.21
nad1	-	936	49.6	7.6	27.2	15.6	76.8	23.2	65.2	-0.291	0.346
nad2	+	1002	46.7	11.5	35.9	5.9	82.6	17.4	52.6	-0.13	-0.322
nad3	+	354	44.1	14.7	34.7	6.5	78.8	21.2	50.6	-0.118	-0.387
nad4	-	1346	50	6	30.6	13.4	80.6	19.4	63.4	-0.241	0.379
nad4L	-	288	53.1	4.2	29.5	13.2	82.6	17.4	66.3	-0.286	0.52
nad5	-	1743	48.5	6.6	31.3	13.6	79.8	20.2	62.1	-0.215	0.347

nad6	+	531	43.3	9.2	42	5.5	85.3	14.7	48.8	-0.015	-0.256
rrnL	-	1373	42.2	4.9	39.9	13	82.1	17.9	55.2	-0.028	0.455
rrnS	-	808	42.8	4.8	42.7	9.7	85.5	14.5	52.5	-0.001	0.333
rRNAs	-	2181	42.4	4.9	40.9	11.8	83.3	16.7	54.2	-0.018	0.416
tRNAs	+	925	38.7	9.1	42.7	9.5	81.4	18.6	48.2	0.049	0.023
tRNAs	-	537	40.6	5	39.5	14.9	80.1	19.9	55.5	-0.014	0.495
Full genome	+	15330	37.5	13.4	41.2	7.8	78.7	21.2	45.3	0.047	-0.265

Table S2.10 The nucleotide composition of PCGs in *Paralebeda femorata*

Regions	Strand	Size (bp)	T(U)	C	A	G	AT(%)	GC(%)	GT(%)	AT skew	GC skew
PCGs	+	6903	41.1	13.9	35.3	9.8	76.4	23.7	50.9	-0.076	-0.172
PCGs	-	4326	48.3	6.5	31.4	13.8	79.7	20.3	62.1	-0.212	0.359
1st codon position	+	2301	33	12.9	38.4	15.7	71.4	28.6	48.7	0.075	0.099
1st codon position	-	1442	41.6	5.6	34	18.7	75.6	24.3	60.3	-0.1	0.538
2nd codon position	+	2301	46.2	18.8	22.6	12.4	68.8	31.2	58.6	-0.343	-0.206
2nd codon position	-	1442	51	13	20.8	15.3	71.8	28.3	66.3	-0.42	0.081
3rd codon position	+	2301	43.9	9.9	44.9	1.3	88.8	11.2	45.2	0.01	-0.767
3rd codon position	-	1442	52.2	1	39.3	7.5	91.5	8.5	59.7	-0.141	0.77
atp6	+	678	42.2	14.9	35.3	7.7	77.5	22.6	49.9	-0.09	-0.32
atp8	+	174	42	8.6	47.7	1.7	89.7	10.3	43.7	0.064	-0.667
cox1	+	1539	39.7	15	31.3	14	71	29	53.7	-0.119	-0.034
cox2	+	682	38.1	14.5	36.5	10.9	74.6	25.4	49	-0.022	-0.145
cox3	+	789	38.8	15.2	33.5	12.5	72.3	27.7	51.3	-0.074	-0.096
cytb	+	1149	40.3	15.1	34.6	10.1	74.9	25.2	50.4	-0.077	-0.197
nad1	-	951	48.4	7.8	28.8	15	77.2	22.8	63.4	-0.253	0.318
nad2	+	1014	46.5	10.1	37.1	6.3	83.6	16.4	52.8	-0.113	-0.229
nad3	+	351	43	14.5	36.2	6.3	79.2	20.8	49.3	-0.086	-0.397
nad4	-	1340	47.6	6.3	31.8	14.3	79.4	20.6	61.9	-0.199	0.384
nad4L	-	294	53.1	3.7	28.6	14.6	81.7	18.3	67.7	-0.3	0.593
nad5	-	1743	47.9	6.4	33	12.7	80.9	19.1	60.6	-0.184	0.327

nad6	+	528	40.3	12.3	41.7	5.7	82	18	46	0.016	-0.368
rrnL	-	1372	43.6	4.7	40.7	11	84.3	15.7	54.6	-0.035	0.398
rrnS	-	797	42.4	4.6	43.4	9.5	85.8	14.1	51.9	0.012	0.345
rRNAs	-	2169	43.2	4.7	41.7	10.5	84.9	15.2	53.7	-0.017	0.38
tRNAs	+	945	38.8	9.4	41.7	10.1	80.5	19.5	48.9	0.035	0.033
tRNAs	-	533	41.1	5.1	39.2	14.6	80.3	19.7	55.7	-0.023	0.486
Full genome	+	15376	38.5	12.9	40.9	7.8	79.4	20.7	46.3	0.03	-0.247

Table S2.11 The nucleotide composition of PCGs in *Sphragifera sigillata*

Regions	Strand	Size (bp)	T(U)	C	A	G	AT(%)	GC(%)	GT(%)	AT skew	GC skew
PCGs	+	6900	44.7	11.6	34.1	9.7	78.8	21.3	54.4	-0.135	-0.089
PCGs	-	4305	48.8	6.2	33.5	11.5	82.3	17.7	60.3	-0.185	0.294
1st codon position	+	2300	35.6	11.7	36.8	15.9	72.4	27.6	51.5	0.017	0.15
1st codon position	-	1435	41	5.9	36.7	16.4	77.7	22.3	57.4	-0.056	0.47
2nd codon position	+	2300	46.3	18.7	22.6	12.5	68.9	31.2	58.8	-0.345	-0.198
2nd codon position	-	1435	51.6	12.3	21.1	15	72.7	27.3	66.6	-0.42	0.1
3rd codon position	+	2300	52.3	4.3	42.8	0.7	95.1	5	53	-0.099	-0.735
3rd codon position	-	1435	53.7	0.6	42.8	2.9	96.5	3.5	56.6	-0.113	0.68
atp6	+	678	44.8	13.4	33.8	8	78.6	21.4	52.8	-0.141	-0.255
atp8	+	162	46.3	6.8	44.4	2.5	90.7	9.3	48.8	-0.02	-0.467
cox1	+	1539	41.1	13.9	31.6	13.3	72.7	27.2	54.4	-0.13	-0.021
cox2	+	682	43.1	11.4	35.3	10.1	78.4	21.5	53.2	-0.099	-0.061
cox3	+	789	43.6	11.9	31.6	12.9	75.2	24.8	56.5	-0.16	0.041
cytb	+	1149	43.3	12.2	34.3	10.2	77.6	22.4	53.5	-0.117	-0.089
nad1	-	939	49.3	7.5	30.8	12.5	80.1	20	61.8	-0.231	0.251
nad2	+	1014	50.8	8.4	34.7	6.1	85.5	14.5	56.9	-0.188	-0.156
nad3	+	357	48.2	10.9	33.9	7	82.1	17.9	55.2	-0.174	-0.219
nad4	-	1340	48.3	6.2	34.2	11.3	82.5	17.5	59.6	-0.171	0.294
nad4L	-	291	54.6	3.8	28.5	13.1	83.1	16.9	67.7	-0.314	0.551
nad5	-	1737	47.9	6	35.3	10.7	83.2	16.7	58.6	-0.151	0.278

nad6	+	531	47.3	8.5	38.8	5.5	86.1	14	52.8	-0.098	-0.216
rrnL	-	973	39.6	6.2	43.9	10.4	83.5	16.6	50	0.052	0.255
rrnS	-	840	42.4	4.9	43.8	8.9	86.2	13.8	51.3	0.017	0.293
rRNAs	-	1813	40.9	5.6	43.8	9.7	84.7	15.3	50.6	0.035	0.271
tRNAs	+	942	39.8	8.7	41.9	9.6	81.7	18.3	49.4	0.026	0.047
tRNAs	-	536	39	5	44	11.9	83	16.9	50.9	0.061	0.407
Full genome	+	15377	41.3	10.8	40.3	7.6	81.6	18.4	48.9	-0.013	-0.176

Table S2.12 The nucleotide composition of PCGs in *Lassaba albidaria*

**Regions**	**Strand**	**Size (bp)**	**T(U)**	**C**	**A**	**G**	**AT(%)**	**GC(%)**	**GT(%)**	**AT skew**	**GC skew**
PCGs	+	6924	41.7	7.5	39.6	11.1	81.3	18.6	52.8	-0.027	0.193
PCGs	-	4350	38.1	11.3	42.1	8.5	80.2	19.8	46.6	0.05	-0.144
1st codon position	+	2308	40.9	5.7	43	10.4	83.9	16.1	51.3	0.026	0.29
1st codon position	-	1450	34.9	12.5	39.9	12.7	74.8	25.2	47.6	0.067	0.008
2nd codon position	+	2308	40.2	8.8	38.4	12.5	78.6	21.3	52.7	-0.023	0.172
2nd codon position	-	1450	41.3	12.4	40.2	6.1	81.5	18.5	47.4	-0.014	-0.343
3rd codon position	+	2308	44.2	8.1	37.3	10.5	81.5	18.6	54.7	-0.085	0.133
3rd codon position	-	1450	38.1	9	46.2	6.6	84.3	15.6	44.7	0.096	-0.154
atp6	+	678	39.4	8.6	41.7	10.3	81.1	18.9	49.7	0.029	0.094
atp8	+	165	33.9	15.8	40	10.3	73.9	26.1	44.2	0.082	-0.209
cox1	+	1545	44.9	7.7	34.4	13	79.3	20.7	57.9	-0.133	0.256
cox2	+	702	37.9	7.8	42.7	11.5	80.6	19.3	49.4	0.06	0.191
cox3	+	789	45.1	5.1	40.9	8.9	86	14	54	-0.049	0.273
cytb	+	1152	37.1	8.4	43.3	11.2	80.4	19.6	48.3	0.078	0.142
nad1	-	945	40.8	12.4	34.3	12.5	75.1	24.9	53.3	-0.087	0.004
nad2	+	1002	43.5	3.6	42.6	10.3	86.1	13.9	53.8	-0.01	0.482
nad3	+	357	52.1	6.4	31.7	9.8	83.8	16.2	61.9	-0.244	0.207
nad4	-	1359	41.4	9.7	42	6.8	83.4	16.5	48.2	0.007	-0.173
nad4L	-	291	41.6	13.7	33.3	11.3	74.9	25	52.9	-0.11	-0.096
nad5	-	1755	33.5	11.6	47.9	7.1	81.4	18.7	40.6	0.176	-0.242

nad6	+	534	37.8	12.7	37.1	12.4	74.9	25.1	50.2	-0.01	-0.015
rrnL	-	1379	43.1	10.8	36.3	9.8	79.4	20.6	52.9	-0.087	-0.049
rrnS	-	811	43.6	8.4	41.9	6	85.5	14.4	49.6	-0.02	-0.162
rRNAs	-	2190	43.3	9.9	38.4	8.4	81.7	18.3	51.7	-0.061	-0.082
tRNAs	+	935	44	7.9	35.8	12.3	79.8	20.2	56.3	-0.102	0.217
tRNAs	-	538	42.9	9.3	39.6	8.2	82.5	17.5	51.1	-0.041	-0.064
Full genome	+	15351	41.4	7.8	39.8	10.9	81.2	18.7	52.3	-0.02	0.165

Table S2.13 The nucleotide composition of PCGs in *Asota tortuosa*

Regions	Strand	Size (bp)	T(U)	C	A	G	AT(%)	GC(%)	GT(%)	AT skew	GC skew
PCGs	+	6897	43.8	12.6	34.1	9.5	77.9	22.1	53.3	-0.124	-0.138
PCGs	-	4308	47.6	6.2	33.3	12.9	80.9	19.1	60.5	-0.177	0.35
1st codon position	+	2299	35.1	12.2	37	15.7	72.1	27.9	50.8	0.027	0.128
1st codon position	-	1436	39.9	6.1	37	17	76.9	23.1	56.9	-0.037	0.474
2nd codon position	+	2299	46.2	18.8	22.8	12.2	69	31	58.4	-0.339	-0.212
2nd codon position	-	1436	51.3	11.9	21.7	15	73	26.9	66.3	-0.405	0.116
3rd codon position	+	2299	50.1	6.7	42.6	0.6	92.7	7.3	50.7	-0.081	-0.844
3rd codon position	-	1436	51.6	0.6	41.2	6.6	92.8	7.2	58.2	-0.113	0.827
atp6	+	678	45	12.8	33.8	8.4	78.8	21.2	53.4	-0.142	-0.208
atp8	+	165	43.6	7.3	45.5	3.6	89.1	10.9	47.2	0.02	-0.333
cox1	+	1536	40.3	14.8	31.4	13.5	71.7	28.3	53.8	-0.124	-0.048
cox2	+	682	41.8	13	35.2	10	77	23	51.8	-0.086	-0.134
cox3	+	789	40.3	13.9	33.5	12.3	73.8	26.2	52.6	-0.093	-0.063
cytb	+	1158	42.8	13.6	33.7	9.9	76.5	23.5	52.7	-0.12	-0.154
nad1	-	936	47.2	7.5	30.3	15	77.5	22.5	62.2	-0.218	0.333
nad2	+	1011	49.7	9.7	35.1	5.5	84.8	15.2	55.2	-0.172	-0.273
nad3	+	354	47.7	11	34.7	6.5	82.4	17.5	54.2	-0.158	-0.258
nad4	-	1340	47.2	5.9	33.7	13.1	80.9	19	60.3	-0.167	0.38
nad4L	-	288	53.8	3.8	31.2	11.1	85	14.9	64.9	-0.265	0.488
nad5	-	1746	47.1	6.1	34.9	11.9	82	18	59	-0.148	0.318

nad6	+	525	48.6	8.8	37.5	5.1	86.1	13.9	53.7	-0.128	-0.26
rrnL	-	1388	41	5	43.2	10.9	84.2	15.9	51.9	0.026	0.373
rrnS	-	809	41.7	4.9	43.1	10.3	84.8	15.2	52	0.017	0.35
rRNAs	-	2197	41.2	5	43.1	10.7	84.3	15.7	51.9	0.023	0.364
tRNAs	+	926	39.2	9.6	41.1	10	80.3	19.6	49.2	0.024	0.022
tRNAs	-	543	39	5.3	43.3	12.3	82.3	17.6	51.3	0.051	0.396
Full genome	+	15385	40.7	11.9	39.9	7.5	80.6	19.4	48.2	-0.011	-0.227

Table S2.14 The nucleotide composition of PCGs in *Hyalinetta circumflexa*

Regions	Strand	Size (bp)	T(U)	C	A	G	AT(%)	GC(%)	GT(%)	AT skew	GC skew
PCGs	+	6918	43.3	11.9	35.4	9.3	78.7	21.2	52.6	-0.1	-0.123
PCGs	-	4308	47.7	6.1	34.2	12	81.9	18.1	59.7	-0.165	0.331
1st codon position	+	2306	35.7	11.7	37.4	15.2	73.1	26.9	50.9	0.023	0.13
1st codon position	-	1436	40.5	5.9	36.5	17.1	77	23	57.6	-0.052	0.486
2nd codon position	+	2306	46.1	18.3	23.5	12.1	69.6	30.4	58.2	-0.323	-0.201
2nd codon position	-	1436	52.5	11.7	21.1	14.7	73.6	26.4	67.2	-0.427	0.113
3rd codon position	+	2306	48.3	5.8	45.4	0.6	93.7	6.4	48.9	-0.031	-0.823
3rd codon position	-	1436	50.1	0.6	45	4.3	95.1	4.9	54.4	-0.054	0.771
atp6	+	678	44	11.9	36.6	7.5	80.6	19.4	51.5	-0.092	-0.227
atp8	+	171	46.8	6.4	43.3	3.5	90.1	9.9	50.3	-0.039	-0.294
cox1	+	1536	40	14.3	32.6	13.2	72.6	27.5	53.2	-0.101	-0.04
cox2	+	682	41.6	11.9	36.7	9.8	78.3	21.7	51.4	-0.064	-0.095
cox3	+	792	41.3	13.1	33.2	12.4	74.5	25.5	53.7	-0.108	-0.03
cytb	+	1155	42.8	13.1	34.4	9.8	77.2	22.9	52.6	-0.109	-0.144
nad1	-	936	47.9	7.4	30.7	14.1	78.6	21.5	62	-0.219	0.313
nad2	+	1014	47.9	9.8	36.5	5.8	84.4	15.6	53.7	-0.136	-0.253
nad3	+	354	48.3	9.9	35.3	6.5	83.6	16.4	54.8	-0.155	-0.207
nad4	-	1338	47.6	6.1	34.1	12.2	81.7	18.3	59.8	-0.166	0.331
nad4L	-	291	51.2	3.8	34	11	85.2	14.8	62.2	-0.202	0.488
nad5	-	1743	47.1	5.7	36.2	11	83.3	16.7	58.1	-0.131	0.32

nad6	+	537	45.6	8.2	41.5	4.7	87.1	12.9	50.3	-0.047	-0.275
rrnL	-	1373	40.6	5	43.9	10.6	84.5	15.6	51.2	0.04	0.362
rrnS	-	785	40.8	4.6	44.1	10.6	84.9	15.2	51.4	0.039	0.395
rRNAs	-	2158	40.6	4.8	44	10.6	84.6	15.4	51.2	0.039	0.373
tRNAs	+	949	40.1	9.1	41.5	9.3	81.6	18.4	49.4	0.017	0.011
tRNAs	-	540	40.6	5	42.4	12	83	17	52.6	0.022	0.413
Full genome	+	15607	41	11.2	40.6	7.3	81.6	18.5	48.3	-0.005	-0.211

Table S2.15 The nucleotide composition of PCGs in *Dolbina inexacta*

Regions	Strand	Size (bp)	T(U)	C	A	G	AT(%)	GC(%)	GT(%)	AT skew	GC skew
PCGs	+	6900	42.6	12.1	35.7	9.6	78.3	21.7	52.2	-0.089	-0.113
PCGs	-	4311	49.7	6.2	32	12.1	81.7	18.3	61.8	-0.216	0.321
1st codon position	+	2300	34.7	11.7	38.4	15.2	73.1	26.9	49.9	0.051	0.128
1st codon position	-	1437	41.8	5.9	36	16.4	77.8	22.3	58.2	-0.074	0.469
2nd codon position	+	2300	46.4	18.5	23	12.1	69.4	30.6	58.5	-0.338	-0.21
2nd codon position	-	1437	52.5	11.8	20.7	14.9	73.2	26.7	67.4	-0.434	0.115
3rd codon position	+	2300	46.7	6	45.7	1.7	92.4	7.7	48.4	-0.011	-0.568
3rd codon position	-	1437	54.8	0.9	39.3	5	94.1	5.9	59.8	-0.164	0.694
atp6	+	678	42.9	12.8	36.3	8	79.2	20.8	50.9	-0.084	-0.234
atp8	+	162	45.7	8	43.8	2.5	89.5	10.5	48.2	-0.021	-0.529
cox1	+	1533	38.7	15	32.7	13.6	71.4	28.6	52.3	-0.083	-0.05
cox2	+	682	41.6	11.6	36.2	10.6	77.8	22.2	52.2	-0.07	-0.046
cox3	+	792	40	13.8	34	12.2	74	26	52.2	-0.082	-0.058
cytb	+	1155	41.6	12.8	35.3	10.2	76.9	23	51.8	-0.082	-0.113
nad1	-	936	49.3	6.8	29.8	14.1	79.1	20.9	63.4	-0.246	0.347
nad2	+	1014	48.7	9.3	36.2	5.8	84.9	15.1	54.5	-0.148	-0.229
nad3	+	354	44.9	10.5	37.9	6.8	82.8	17.3	51.7	-0.085	-0.213
nad4	-	1335	49.7	6.7	31.8	11.9	81.5	18.6	61.6	-0.22	0.282
nad4L	-	291	52.9	3.4	32.6	11	85.5	14.4	63.9	-0.237	0.524
nad5	-	1749	49.4	6	33.3	11.3	82.7	17.3	60.7	-0.195	0.307

nad6	+	531	46.7	7	40.9	5.5	87.6	12.5	52.2	-0.067	-0.121
rrnL	-	1401	42.5	5	41.8	10.8	84.3	15.8	53.3	-0.008	0.367
rrnS	-	812	41.5	4.8	44.1	9.6	85.6	14.4	51.1	0.03	0.333
rRNAs	-	2213	42.1	4.9	42.6	10.3	84.7	15.2	52.4	0.006	0.355
tRNAs	+	933	39	9.2	42.2	9.5	81.2	18.7	48.5	0.04	0.017
tRNAs	-	532	41.2	5.3	41.4	12.2	82.6	17.5	53.4	0.002	0.398
Full genome	+	15232	39.6	11.5	41.3	7.6	80.9	19.1	47.2	0.022	-0.2

Table S2.16 The nucleotide composition of PCGs in *Dolbina paraexacta*

Regions	Strand	Size (bp)	T(U)	C	A	G	AT(%)	GC(%)	GT(%)	AT skew	GC skew
PCGs	+	6900	43	11.6	35.9	9.5	78.9	21.1	52.5	-0.09	-0.1
PCGs	-	4311	49.6	6.3	32.1	12	81.7	18.3	61.6	-0.214	0.311
1st codon position	+	2300	35	11.4	38.4	15.2	73.4	26.6	50.2	0.047	0.142
1st codon position	-	1437	41.7	5.9	36	16.4	77.7	22.3	58.1	-0.073	0.469
2nd codon position	+	2300	46.4	18.5	23	12.1	69.4	30.6	58.5	-0.338	-0.209
2nd codon position	-	1437	52.5	11.9	20.7	15	73.2	26.9	67.5	-0.435	0.114
3rd codon position	+	2300	47.7	4.9	46.3	1.2	94	6.1	48.9	-0.014	-0.612
3rd codon position	-	1437	54.6	1.1	39.6	4.7	94.2	5.8	59.3	-0.159	0.619
atp6	+	678	42.9	12.7	36.6	7.8	79.5	20.5	50.7	-0.08	-0.237
atp8	+	162	45.1	8	44.4	2.5	89.5	10.5	47.6	-0.007	-0.529
cox1	+	1533	39	14.3	33.3	13.4	72.3	27.7	52.4	-0.079	-0.031
cox2	+	682	41.8	11.4	36.8	10	78.6	21.4	51.8	-0.063	-0.068
cox3	+	792	40.9	12.6	34.1	12.4	75	25	53.3	-0.091	-0.01
cytb	+	1155	42.3	12.4	35.5	9.8	77.8	22.2	52.1	-0.088	-0.117
nad1	-	936	48.5	7.5	29.4	14.6	77.9	22.1	63.1	-0.246	0.324
nad2	+	1014	49.1	8.9	36.2	5.8	85.3	14.7	54.9	-0.151	-0.208
nad3	+	354	46	9.3	38.1	6.5	84.1	15.8	52.5	-0.094	-0.179
nad4	-	1335	50	6.4	32	11.6	82	18	61.6	-0.219	0.286
nad4L	-	291	52.6	4.1	32.6	10.7	85.2	14.8	63.3	-0.234	0.442
nad5	-	1749	49.3	5.9	33.6	11.1	82.9	17	60.4	-0.19	0.304

nad6	+	531	46.7	7.2	40.3	5.8	87	13	52.5	-0.074	-0.101
rrnL	-	1391	42.6	5	41.6	10.8	84.2	15.8	53.4	-0.011	0.364
rrnS	-	777	42.3	4.5	43.5	9.7	85.8	14.2	52	0.013	0.364
rRNAs	-	2168	42.5	4.8	42.3	10.4	84.8	15.2	52.9	-0.002	0.364
tRNAs	+	935	39.4	8.8	42.4	9.5	81.8	18.3	48.9	0.037	0.041
tRNAs	-	532	40.8	5.1	41.4	12.8	82.2	17.9	53.6	0.007	0.432
Full genome	+	15220	39.8	11.2	41.4	7.6	81.2	18.8	47.4	0.02	-0.192

Table S2.17 The nucleotide composition of PCGs in *Olivenebula oberthueri*

Regions	Strand	Size (bp)	T(U)	C	A	G	AT(%)	GC(%)	GT(%)	AT skew	GC skew
PCGs	+	6903	42.5	13.4	33.9	10.2	76.4	23.6	52.7	-0.113	-0.137
PCGs	-	4314	47.4	6.6	32.8	13.2	80.2	19.8	60.6	-0.182	0.336
1st codon position	+	2301	34.8	12.6	36.3	16.3	71.1	28.9	51.1	0.021	0.129
1st codon position	-	1438	40	6.3	36.5	17.2	76.5	23.5	57.2	-0.045	0.467
2nd codon position	+	2301	46.2	18.9	22.6	12.3	68.8	31.2	58.5	-0.343	-0.209
2nd codon position	-	1438	51.5	12.2	21.1	15.2	72.6	27.4	66.7	-0.42	0.107
3rd codon position	+	2301	46.6	8.8	42.8	1.9	89.4	10.7	48.5	-0.043	-0.649
3rd codon position	-	1438	50.7	1.3	40.8	7.3	91.5	8.6	58	-0.109	0.707
atp6	+	678	43.2	14.3	34.4	8.1	77.6	22.4	51.3	-0.114	-0.276
atp8	+	162	46.3	5.6	45.1	3.1	91.4	8.7	49.4	-0.014	-0.286
cox1	+	1536	39.9	15	31	14.1	70.9	29.1	54	-0.126	-0.029
cox2	+	682	39.3	14.2	36.2	10.3	75.5	24.5	49.6	-0.041	-0.162
cox3	+	789	39.2	15.2	31.6	14.1	70.8	29.3	53.3	-0.108	-0.039
cytb	+	1152	41.4	14.3	33.8	10.5	75.2	24.8	51.9	-0.102	-0.154
nad1	-	939	47.7	7.7	29.5	15.1	77.2	22.8	62.8	-0.236	0.327
nad2	+	1014	48.8	10.5	34.1	6.6	82.9	17.1	55.4	-0.177	-0.225
nad3	+	357	46.5	11.2	35.6	6.7	82.1	17.9	53.2	-0.133	-0.25
nad4	-	1340	46.9	6.5	33.9	12.8	80.8	19.3	59.7	-0.161	0.326
nad4L	-	291	53.3	4.1	29.9	12.7	83.2	16.8	66	-0.281	0.51
nad5	-	1746	46.7	6.5	34.2	12.7	80.9	19.2	59.4	-0.154	0.323

nad6	+	534	44.9	11.6	37.3	6.2	82.2	17.8	51.1	-0.093	-0.305
rrnL	-	1354	41.1	4.9	42.3	11.7	83.4	16.6	52.8	0.014	0.411
rrnS	-	814	41.9	4.9	43.5	9.7	85.4	14.6	51.6	0.019	0.328
rRNAs	-	2168	41.4	4.9	42.8	10.9	84.2	15.8	52.3	0.016	0.382
tRNAs	+	939	39.7	9.4	41.1	9.8	80.8	19.2	49.5	0.017	0.022
tRNAs	-	529	39.7	5.7	42.2	12.5	81.9	18.2	52.2	0.03	0.375
Full genome	+	15457	39.8	12.4	39.9	7.9	79.7	20.3	47.7	0	-0.22

Table S3.1 The characteristics of coding amino acids in *Zaranga tukuringra*

AA	Count	%
Phe(F)	366	9.84
Leu2(L2)	454	12.2
Leu1(L1)	103	2.77
Ile(I)	448	12.04
Met(M)	283	7.61
Val(V)	145	3.9
Ser2(S2)	201	5.4
Pro(P)	126	3.39
Thr(T)	162	4.35
Ala(A)	127	3.41
Tyr(Y)	188	5.05
His(H)	67	1.8
Gln(Q)	62	1.67
Asn(N)	252	6.77
Lys(K)	101	2.71
Asp(D)	65	1.75
Glu(E)	73	1.96
Cys(C)	31	0.83
Trp(W)	96	2.58
Arg(R)	53	1.42
Ser1(S1)	119	3.2
Gly(G)	199	5.35
codon end in A or T	3289	88.39
codon end in G or T	1944	52.24
Total	3721	

Table S3.2 The characteristics of coding amino acids in *Gazalina chrysolopha*

AA	Count	%
Phe(F)	358	9.58
Leu2(L2)	421	11.26
Leu1(L1)	137	3.67
Ile(I)	423	11.32
Met(M)	280	7.49
Val(V)	178	4.76
Ser2(S2)	202	5.4
Pro(P)	134	3.58
Thr(T)	162	4.33
Ala(A)	125	3.34
Tyr(Y)	181	4.84
His(H)	71	1.9
Gln(Q)	65	1.74
Asn(N)	253	6.77
Lys(K)	104	2.78
Asp(D)	65	1.74
Glu(E)	73	1.95
Cys(C)	33	0.88
Trp(W)	96	2.57
Arg(R)	53	1.42
Ser1(S1)	118	3.16
Gly(G)	206	5.51
codon end in A or T	3007	80.44
codon end in G or T	1884	50.4
Total	3738	

Table S3.3 The characteristics of coding amino acids in *Deroca hyalina*

AA	Count	%
Phe(F)	370	9.9
Leu2(L2)	494	13.22
Leu1(L1)	50	1.34
Ile(I)	459	12.28
Met(M)	317	8.48
Val(V)	128	3.42
Ser2(S2)	209	5.59
Pro(P)	122	3.26
Thr(T)	147	3.93
Ala(A)	124	3.32
Tyr(Y)	187	5
His(H)	68	1.82
Gln(Q)	67	1.79
Asn(N)	266	7.12
Lys(K)	112	3
Asp(D)	63	1.69
Glu(E)	74	1.98
Cys(C)	31	0.83
Trp(W)	95	2.54
Arg(R)	53	1.42
Ser1(S1)	112	3
Gly(G)	190	5.08
codon end in A or T	3515	94.03
codon end in G or T	1969	52.68
Total	3738	

Table S3.4 The characteristics of coding amino acids in *Menophra sp.*

AA	Count	%
Phe(F)	359	9.61
Leu2(L2)	529	14.17
Leu1(L1)	43	1.15
Ile(I)	455	12.19
Met(M)	271	7.26
Val(V)	151	4.04
Ser2(S2)	205	5.49
Pro(P)	122	3.27
Thr(T)	144	3.86
Ala(A)	128	3.43
Tyr(Y)	200	5.36
His(H)	67	1.79
Gln(Q)	60	1.61
Asn(N)	262	7.02
Lys(K)	105	2.81
Asp(D)	67	1.79
Glu(E)	74	1.98
Cys(C)	34	0.91
Trp(W)	96	2.57
Arg(R)	53	1.42
Ser1(S1)	113	3.03
Gly(G)	196	5.25
codon end in A or T	3485	93.33
codon end in G or T	1998	53.51
Total	3734	

Table S3.5 The characteristics of coding amino acids in *Dolbina paraexacta*

AA	Count	%
Phe(F)	377	10.12
Leu2(L2)	502	13.48
Leu1(L1)	42	1.13
Ile(I)	468	12.56
Met(M)	303	8.13
Val(V)	130	3.49
Ser2(S2)	199	5.34
Pro(P)	126	3.38
Thr(T)	149	4
Ala(A)	122	3.28
Tyr(Y)	186	4.99
His(H)	67	1.8
Gln(Q)	61	1.64
Asn(N)	259	6.95
Lys(K)	105	2.82
Asp(D)	65	1.74
Glu(E)	71	1.91
Cys(C)	30	0.81
Trp(W)	97	2.6
Arg(R)	52	1.4
Ser1(S1)	117	3.14
Gly(G)	197	5.29
codon end in A or T	3514	94.34
codon end in G or T	1975	53.02
Total	3725	

Table S3.6 The characteristics of coding amino acids in *Lassaba albidaria*

AA	Count	%
Phe(F)	337	9.72
Leu2(L2)	325	9.37
Leu1(L1)	98	2.83
Ile(I)	361	10.41
Met(M)	274	7.9
Val(V)	132	3.81
Ser2(S2)	151	4.35
Pro(P)	66	1.9
Thr(T)	106	3.06
Ala(A)	61	1.76
Tyr(Y)	210	6.06
His(H)	59	1.7
Gln(Q)	59	1.7
Asn(N)	356	10.27
Lys(K)	340	9.8
Asp(D)	73	2.1
Glu(E)	83	2.39
Cys(C)	50	1.44
Trp(W)	86	2.48
Arg(R)	31	0.89
Ser1(S1)	135	3.89
Gly(G)	75	2.16
codon end in A or T	3102	89.45
codon end in G or T	1911	55.1
Total	3468	

Table S3.7 The characteristics of coding amino acids in *Sphragifera sigillata*

AA	Count	%
Phe(F)	376	10.1
Leu2(L2)	493	13.24
Leu1(L1)	49	1.32
Ile(I)	479	12.86
Met(M)	269	7.22
Val(V)	140	3.76
Ser2(S2)	209	5.61
Pro(P)	125	3.36
Thr(T)	146	3.92
Ala(A)	125	3.36
Tyr(Y)	186	4.99
His(H)	67	1.8
Gln(Q)	61	1.64
Asn(N)	254	6.82
Lys(K)	106	2.85
Asp(D)	65	1.75
Glu(E)	72	1.93
Cys(C)	32	0.86
Trp(W)	99	2.66
Arg(R)	53	1.42
Ser1(S1)	119	3.2
Gly(G)	199	5.34
codon end in A or T	3572	95.92
codon end in G or T	2030	54.51
Total	3724	

Table S3.8 The characteristics of coding amino acids in *Asota tortuosa*

AA	Count	%
Phe(F)	357	9.59
Leu2(L2)	476	12.78
Leu1(L1)	62	1.66
Ile(I)	471	12.65
Met(M)	293	7.87
Val(V)	140	3.76
Ser2(S2)	209	5.61
Pro(P)	126	3.38
Thr(T)	143	3.84
Ala(A)	125	3.36
Tyr(Y)	199	5.34
His(H)	64	1.72
Gln(Q)	62	1.66
Asn(N)	251	6.74
Lys(K)	106	2.85
Asp(D)	67	1.8
Glu(E)	76	2.04
Cys(C)	32	0.86
Trp(W)	95	2.55
Arg(R)	53	1.42
Ser1(S1)	119	3.2
Gly(G)	198	5.32
codon end in A or T	3464	93.02
codon end in G or T	2001	53.73
Total	3724	

Table S3.9 The characteristics of coding amino acids in *Olivenebula oberthueri*

AA	Count	%
Phe(F)	366	9.82
Leu2(L2)	478	12.82
Leu1(L1)	73	1.96
Ile(I)	455	12.2
Met(M)	277	7.43
Val(V)	155	4.16
Ser2(S2)	204	5.47
Pro(P)	125	3.35
Thr(T)	151	4.05
Ala(A)	130	3.49
Tyr(Y)	190	5.1
His(H)	67	1.8
Gln(Q)	62	1.66
Asn(N)	244	6.55
Lys(K)	107	2.87
Asp(D)	68	1.82
Glu(E)	74	1.98
Cys(C)	29	0.78
Trp(W)	97	2.6
Arg(R)	53	1.42
Ser1(S1)	126	3.38
Gly(G)	197	5.28
codon end in A or T	3371	90.42
codon end in G or T	1949	52.28
Total	3728	

Table S3.10 The characteristics of coding amino acids in *Psyra falcipennis*

AA	Count	%
Phe(F)	358	9.61
Leu2(L2)	511	13.71
Leu1(L1)	38	1.02
Ile(I)	443	11.89
Met(M)	287	7.7
Val(V)	153	4.11
Ser2(S2)	204	5.47
Pro(P)	121	3.25
Thr(T)	152	4.08
Ala(A)	128	3.43
Tyr(Y)	199	5.34
His(H)	66	1.77
Gln(Q)	58	1.56
Asn(N)	266	7.14
Lys(K)	106	2.84
Asp(D)	65	1.74
Glu(E)	75	2.01
Cys(C)	37	0.99
Trp(W)	96	2.58
Arg(R)	53	1.42
Ser1(S1)	113	3.03
Gly(G)	198	5.31
codon end in A or T	3486	93.53
codon end in G or T	1985	53.26
Total	3727	

Table S3.11 The characteristics of coding amino acids in *Numenes albofascia*

AA	Count	%
Phe(F)	309	9.78
Leu2(L2)	399	12.63
Leu1(L1)	78	2.47
Ile(I)	377	11.94
Met(M)	228	7.22
Val(V)	140	4.43
Ser2(S2)	186	5.89
Pro(P)	106	3.36
Thr(T)	112	3.55
Ala(A)	102	3.23
Tyr(Y)	157	4.97
His(H)	59	1.87
Gln(Q)	47	1.49
Asn(N)	204	6.46
Lys(K)	88	2.79
Asp(D)	52	1.65
Glu(E)	71	2.25
Cys(C)	28	0.89
Trp(W)	82	2.6
Arg(R)	45	1.42
Ser1(S1)	115	3.64
Gly(G)	173	5.48
codon end in A or T	2749	87.05
codon end in G or T	1687	53.42
Total	3158	

Table S3.12 The characteristics of coding amino acids in *Rhagastis albomarginatus*

AA	Count	%
Phe(F)	322	9.65
Leu2(L2)	445	13.33
Leu1(L1)	50	1.5
Ile(I)	415	12.43
Met(M)	248	7.43
Val(V)	117	3.51
Ser2(S2)	176	5.27
Pro(P)	119	3.57
Thr(T)	146	4.37
Ala(A)	101	3.03
Tyr(Y)	186	5.57
His(H)	62	1.86
Gln(Q)	58	1.74
Asn(N)	237	7.1
Lys(K)	95	2.85
Asp(D)	53	1.59
Glu(E)	68	2.04
Cys(C)	27	0.81
Trp(W)	89	2.67
Arg(R)	48	1.44
Ser1(S1)	98	2.94
Gly(G)	178	5.33
codon end in A or T	3083	92.36
codon end in G or T	1702	50.99
Total	3338	

Table S3.13 The characteristics of coding amino acids in *Dolbina inexacta*

AA	Count	%
Phe(F)	379	10.17
Leu2(L2)	500	13.42
Leu1(L1)	48	1.29
Ile(I)	467	12.54
Met(M)	299	8.03
Val(V)	130	3.49
Ser2(S2)	195	5.23
Pro(P)	126	3.38
Thr(T)	152	4.08
Ala(A)	123	3.3
Tyr(Y)	185	4.97
His(H)	68	1.83
Gln(Q)	61	1.64
Asn(N)	259	6.95
Lys(K)	105	2.82
Asp(D)	65	1.74
Glu(E)	71	1.91
Cys(C)	30	0.81
Trp(W)	97	2.6
Arg(R)	52	1.4
Ser1(S1)	118	3.17
Gly(G)	195	5.23
codon end in A or T	3476	93.32
codon end in G or T	1971	52.91
Total	3725	

Table S3.14 The characteristics of coding amino acids in *Paralebeda femorata*

AA	Count	%
Phe(F)	368	9.86
Leu2(L2)	456	12.22
Leu1(L1)	72	1.93
Ile(I)	426	11.41
Met(M)	310	8.31
Val(V)	166	4.45
Ser2(S2)	211	5.65
Pro(P)	121	3.24
Thr(T)	164	4.39
Ala(A)	124	3.32
Tyr(Y)	189	5.06
His(H)	67	1.8
Gln(Q)	64	1.71
Asn(N)	252	6.75
Lys(K)	100	2.68
Asp(D)	64	1.71
Glu(E)	73	1.96
Cys(C)	28	0.75
Trp(W)	97	2.6
Arg(R)	53	1.42
Ser1(S1)	123	3.3
Gly(G)	204	5.47
codon end in A or T	3363	90.11
codon end in G or T	1902	50.96
Total	3732	

Table S3.15 The characteristics of coding amino acids in *Zeuzera pyrina*

AA	Count	%
Phe(F)	363	9.71
Leu2(L2)	476	12.74
Leu1(L1)	98	2.62
Ile(I)	454	12.15
Met(M)	256	6.85
Val(V)	160	4.28
Ser2(S2)	214	5.73
Pro(P)	126	3.37
Thr(T)	157	4.2
Ala(A)	127	3.4
Tyr(Y)	184	4.92
His(H)	66	1.77
Gln(Q)	64	1.71
Asn(N)	234	6.26
Lys(K)	104	2.78
Asp(D)	67	1.79
Glu(E)	74	1.98
Cys(C)	32	0.86
Trp(W)	98	2.62
Arg(R)	50	1.34
Ser1(S1)	134	3.59
Gly(G)	199	5.33
codon end in A or T	3317	88.76
codon end in G or T	1974	52.82
Total	3737	

Table S3.16 The characteristics of coding amino acids in *Phyllosphingia dissimilis*

**AA**	**Count**	**%**
Phe(F)	385	10.32
Leu2(L2)	505	13.54
Leu1(L1)	45	1.21
Ile(I)	466	12.49
Met(M)	284	7.61
Val(V)	133	3.56
Ser2(S2)	203	5.44
Pro(P)	121	3.24
Thr(T)	144	3.86
Ala(A)	122	3.27
Tyr(Y)	207	5.55
His(H)	66	1.77
Gln(Q)	64	1.72
Asn(N)	263	7.05
Lys(K)	106	2.84
Asp(D)	62	1.66
Glu(E)	72	1.93
Cys(C)	31	0.83
Trp(W)	93	2.49
Arg(R)	53	1.42
Ser1(S1)	110	2.95
Gly(G)	196	5.25
codon end in A or T	3576	95.85
codon end in G or T	2023	54.22
Total	3731	

Table S3.17 The characteristics of coding amino acids in *Hyalinetta circumflexa*

AA	Count	%
Phe(F)	363	9.73
Leu2(L2)	500	13.4
Leu1(L1)	48	1.29
Ile(I)	461	12.36
Met(M)	303	8.12
Val(V)	141	3.78
Ser2(S2)	196	5.25
Pro(P)	124	3.32
Thr(T)	145	3.89
Ala(A)	124	3.32
Tyr(Y)	207	5.55
His(H)	70	1.88
Gln(Q)	60	1.61
Asn(N)	259	6.94
Lys(K)	104	2.79
Asp(D)	61	1.64
Glu(E)	73	1.96
Cys(C)	32	0.86
Trp(W)	94	2.52
Arg(R)	53	1.42
Ser1(S1)	114	3.06
Gly(G)	198	5.31
codon end in A or T	3525	94.5
codon end in G or T	1908	51.15
Total	3730	
